# IGHV allele similarity clustering improves genotype inference from adaptive immune receptor repertoire sequencing data

**DOI:** 10.1093/nar/gkad603

**Published:** 2023-08-07

**Authors:** Ayelet Peres, William D Lees, Oscar L Rodriguez, Noah Y Lee, Pazit Polak, Ronen Hope, Meirav Kedmi, Andrew M Collins, Mats Ohlin, Steven H Kleinstein, Corey T Watson, Gur Yaari

**Affiliations:** Faculty of Engineering, Bar Ilan University, 5290002 Ramat Gan, Israel; Bar Ilan Institute of Nanotechnology and Advanced Materials, Bar Ilan University, 5290002 Ramat Gan, Israel; Institute of Structural and Molecular Biology, Birkbeck College, University of London, London, WC1E 7JE, UK; Department of Biochemistry and Molecular Genetics, University of Louisville School of Medicine, Louisville, KY, 40202, USA; Program in Computational Biology & Bioinformatics, Yale University, New Haven, CT, 06511, USA; Department of Pathology, Yale School of Medicine, New Haven, CT, 06520, USA; Faculty of Engineering, Bar Ilan University, 5290002 Ramat Gan, Israel; Bar Ilan Institute of Nanotechnology and Advanced Materials, Bar Ilan University, 5290002 Ramat Gan, Israel; Faculty of Engineering, Bar Ilan University, 5290002 Ramat Gan, Israel; Department of Pathology, Yale School of Medicine, New Haven, CT, 06520, USA; Division of Hematology and Bone Marrow Transplantation, Chaim Sheba Medical Center, Tel-Hashomer, 5262000, Israel; Sackler School of Medicine, Tel-Aviv University, Tel-Aviv, 69978, Israel; School of Biotechnology and Biomedical Sciences, University of New South Wales, Sydney, NSW 2052, Australia; Department of Immunotechnology Lund University, Lund, 221 00, Sweden; Program in Computational Biology & Bioinformatics, Yale University, New Haven, CT, 06511, USA; Department of Pathology, Yale School of Medicine, New Haven, CT, 06520, USA; Department of Biochemistry and Molecular Genetics, University of Louisville School of Medicine, Louisville, KY, 40202, USA; Faculty of Engineering, Bar Ilan University, 5290002 Ramat Gan, Israel; Bar Ilan Institute of Nanotechnology and Advanced Materials, Bar Ilan University, 5290002 Ramat Gan, Israel

## Abstract

In adaptive immune receptor repertoire analysis, determining the germline variable (V) allele associated with each T- and B-cell receptor sequence is a crucial step. This process is highly impacted by allele annotations. Aligning sequences, assigning them to specific germline alleles, and inferring individual genotypes are challenging when the repertoire is highly mutated, or sequence reads do not cover the whole V region. Here, we propose an alternative naming scheme for the V alleles, as well as a novel method to infer individual genotypes. We demonstrate the strengths of the two by comparing their outcomes to other genotype inference methods. We validate the genotype approach with independent genomic long-read data. The naming scheme is compatible with current annotation tools and pipelines. Analysis results can be converted from the proposed naming scheme to the nomenclature determined by the International Union of Immunological Societies (IUIS). Both the naming scheme and the genotype procedure are implemented in a freely available R package (PIgLET https://bitbucket.org/yaarilab/piglet). To allow researchers to further explore the approach on real data and to adapt it for their uses, we also created an interactive website (https://yaarilab.github.io/IGHV_reference_book).

## Graphical Abstract

**Graphical Abstract:**
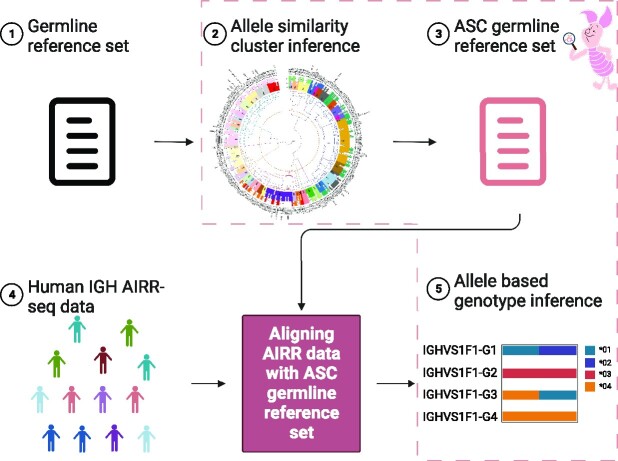
Created with BioRender.com

## INTRODUCTION

The adaptive immune system is key in fighting the diverse array of pathogens our bodies encounter. Adaptive immune receptor repertoire sequencing (AIRR-seq) is a rising approach for studying the dynamics of immune responses (1). Two crucial steps in AIRR-seq analyses are the inference of germline variable (V), diversity (D) and joining (J) allele sequences and, in B cells, the inference of clonal lineages. The immunoglobulin (Ig)-encoding genomic loci are challenging to study because of their repetitive nature and structural variants (2–4). The complex structure of the human Ig heavy chain V (IGHV) locus on chromosome 14 is illustrated in Supplementary Figure S1A.

A widely used taxonomy for human IG genes, which provides a common language for V, D, and J germline subgroups, genes, and alleles (2,5), was codified by the ImMunoGeneTics Information System (IMGT) (6). This nomenclature is referred to here as the International Union of Immunological Societies (IUIS) nomenclature, for the gene names are allocated according to a process governed by the IUIS. With recent technological and algorithmic advances in the field, many previously unknown alleles and structural variants have been reported (4,7–11). Naming these variants poses a challenge on the IUIS naming scheme, as it is not always clear how to link a germline allele sequence to a specific gene.

Germline annotation is typically performed using an aligner tool, which determines the germline allele by comparison to sequences listed in a ‘germline reference set’. For V genes, the accuracy of this assignment is strongly influenced by the sequencing coverage (9,12). Sequencing protocols that span all 290-320 base pairs of the V sequence permit the greatest accuracy, but partial coverage sequencing is often employed. Two such common protocols are BIOMED-2 (13), which utilizes primers in the framework 1 (FW1) and framework 2 (FW2) regions, and ImmunoSeq (14), which amplifies and resolves only the complementarity-determining region 3 (CDR3) and a small fragment of the V and J regions. Such partial coverage of the V region dramatically reduces the number of alleles that can be categorically resolved. This is particularly problematic as there is reduced diversity at the 3′ end of the V gene germline sequences (9,15,16). Even when the sequences span the whole V region, sequence alignment against the germline reference set often does not resolve a single categorical germline allele for every sequence, because of duplicated sequences within the germline reference set itself. These duplicated sequences within the germline reference set reflect known gene duplications in the locus, and the fact that identical allele sequences can be localized to more than one gene in different individuals. In addition, B cells undergo an affinity maturation process that involves somatic hypermutations, which result in mismatches from the germline. This can cause a V sequence to become equidistant from more than one sequence in the set , again leading to multiple allelic assignments. Inference of a personal genotype is an important step, in which the set of V(D)J alleles an individual carries is inferred from the set of sequence annotations in a repertoire (17–19). This step reduces the level of ambiguity for the annotations of individual sequences, by restricting the complete set of alleles to the personal genotype.

Ambiguity in allele assignments hinders clonal inference (12,20). Each clone stems from an ancestral naive B cell expressing an unmutated B cell receptor (BCR). In AIRR-seq analysis, it is common to infer a BCR clone first as a group of sequences that share the same V and J germline assignments and CDR3 length (21), and then cluster the sequences in this group based on similarity in the CDR3 sequence. To achieve correct clonal inference, accurately annotating the AIRR-seq data is therefore crucial, as multiple or mis-assignments can result in biased clonal inference. Moreover, inferring clones based on gene annotations instead of allele annotations may obscure important information.

**Figure 1.**
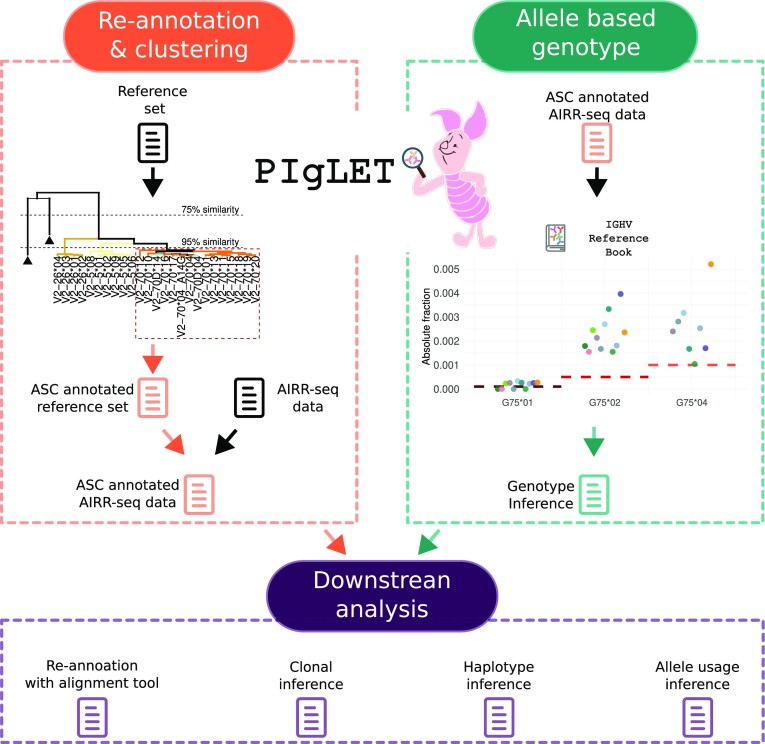
PIgLET workflow The left upper panel illustrates the re-annotation and clustering inference steps. Our Program for Ig Allele Similarity Clusters and Genotypes (PIgLET) receives as input a reference set, and infers clusters using hierarchical clustering based on sequence similarity. Then an ASC annotated reference set is obtained. The next step is re-annotating an AIRR-seq data with the reference set. The output of this process is an ASC annotated AIRR-seq dataset (marked in orange). The right upper panel shows the ASC-based genotyping approach of PIgLET. PIgLET receives as input the ASC annotated AIRR-seq data. The genotype is inferred based on allele-specific threshold from the IGHV reference book. Finally, a genotype inference is generated (marked in green). The bottom panel outlines possible downstream analyses that can be conducted using PIgLET’s output, with improved performance.

To address these challenges, we propose a two component approach, illustrated in Figure 1. We first propose a new naming scheme for IGHV germline sequences. This is followed by a new genotype inference step that is based on the expression of each allele independently in the repertoire.

The current IUIS naming scheme classifies IGHV sequences by subgroup, gene, and allele. Subgroup is determined by sequence similarity and gene is determined by the ostensible order of appearance along the chromosomal locus (2). Since the order of genes along the locus is not well determined and likely varies between individuals, the IUIS names are sub-optimal for many analysis tasks. To address this, our proposed naming scheme is based on the clustering of alleles into Allele Sequence Clusters (ASCs) solely on the basis of similarity. These ASCs take the place of genes in the analysis. The intention here is not to replace the IUIS system in reporting analysis results, but rather to offer a method of data representation that is more tractable for analysis purposes.

The second part of our proposed approach is aimed at addressing the challenge of inferring personal genotypes. Current tools for genotype inference adopt a gene-based approach to determine the presence of an allele. This approach is based on calculating the relative frequency of an allele, which is then normalized by the total number of sequences assigned to the corresponding gene (17,22,23). This method is hindered by allele similarity, as many sequences are assigned to multiple alleles from different genes. Here, we propose a new genotyping approach, based on the consideration of the overall frequency of each allele normalized by the total number of sequences in the whole repertoire. Our method takes advantage of the new proposed naming scheme that collapses identical sequences into a single name. We show that our allele-based genotyping approach provides improved results compared to existing tools and is in excellent correspondence with genotypes derived from genomic sequencing.

## MATERIALS AND METHODS

### Data

We used heavy chain BCR repertoire data, sequenced from naive and non-naive B cells from individuals of three VDJbase (16) projects: P1 (naive), P11 (naive), and P4 (non-naive). Library preparation and processing for projects P1 and P11 were performed as described in (24). The processing for project P4 repertoires is described in (25). For the current study, we downloaded the V, D and J allele reference set from IMGT (imgt.org) on July 2022, including the functionality annotation for each allele. Non-functional alleles from the V reference set were discarded, resulting in the exclusion of subgroup IGHV8, as none of the alleles in this subgroup are functional. Hence, the final V reference set used here included only alleles from subgroups IGHV1–7.

### Allele similarity clusters

To create the allele similarity clusters (ASCs), we used the most recent available IGHV reference set from IMGT, with addition of undocumented allele sequences not found in the IMGT germline reference set, inferred from both P1 and P11. The combined set was then filtered to include only functional alleles that start from the first position of the V sequence region as defined by the IMGT numbering scheme, and extending through at least position 318. This resulted in 280 unique allele sequences. We next trimmed the 3′ ends of any germline sequences longer than 318 bp. Our objective here was to maximize sequence lengths while facilitating accurate comparisons of sequences with equivalent sequence content. Position 318 was seleted as the optimum based on the fact that, in AIRR-seq data, nucleotide variation beyond this position are impossible to reliably detect (8). Following trimming, the germline reference set consisted of 278 unique alleles, which were then used to generate ASCs. For clustering, we calculated the Levenshtein distance between all allele pairs up to position 318, followed by a hierarchical clustering step with complete linkage. The resulting tree was cut based on two similarity thresholds of $75\%$ and $95\%$, to obtain the allele families and ASCs, respectively. In setting the 95% similarity threshold, there was a trade off between the overall number of clusters and the number of clusters that contain alleles from distinct genes. To set the threshold, we plotted two metric evaluation as a function of the similarity threshold. First, the number of genes spanning more than one cluster, normalized by the total number of genes. Second, the number of clusters that contain alleles from distinct genes, normalized by the total number of clusters (Supplementary Figure S2). The plots show that the optimal range for the threshold is between 94% and 96%. For simplicity, we chose 95%, representing the middle of this range. Critically, the optimal threshold may vary depending on the germline reference set used. Thus, when additional human alleles are discovered and incorporated, this threshold may need to be adjusted; additionally, implementation of the ASC approach for other species will require optimization of germline reference set trimming and clustering thresholds depending on the input data.

As a result of the clustering, the alleles were renamed to represent the new allele families and ASCs (Supplementary Table S1). For example, for the allele IGHVS1F2-G15*02, the family is represented by F2, the ASC by G15, and the allele by 02. S1 is an indicator of the library amplicon length of a given reference set. A key table that links between the IUIS naming scheme and the ASC naming scheme can be found in Supplementary Table S1. The reference set with the new naming scheme was then used for downstream processing.

It is worth mentioning a potential issue that concerns all AIRR-seq based genotype inference approaches: in some rare cases, two alleles differ only at the 3′ end of the sequence. In human IGHV, this means beyond 318 in the IMGT numbering scheme. This imposes many instances of multiple assignments, as the aligners cannot differentiate between the two when the rearrangements had been trimmed upstream of the position 318, during the rearrangement process. In human IGHV, only two such cases exist, 3-66*01 and 3-66*04, 4-28*01 and 4-28*03. These cases should be treated separately, considering all particularities of the sequences, and should be reported with an adequate confidence level (Supplementary Table S1). Hence, for the downstream analysis in this paper, to avoid effects on the alignment process, V germline sequences that were trimmed for clustering were extended to their original length. As alleles 01 and 04 of IGHV3-66 and alleles 01 and 03 of IGHV4-28 only differ at position 319, they were collapsed in the cluster analysis. Hence, in the reference set we have decided to include both alleles for both genes.

### ASC-based genotype method

The ASC-based genotype utilizes an empirically derived threshold to determine the presence of a given allele within an individual’s genotype. We first had to set a default threshold (10^−4^) for the absolute allele usage fraction before tailoring it to each allele. The choice of 10^−4^ was based on the current typical depth of AIRR-seq data, which in our datasets was between 10K and 20K sequences. With these depths, the threshold of 10^−4^ includes sequences that appear once or twice in a given sample. We then tuned the individual thresholds, from observations of the allele’s usage across all available naive B cell samples present in VDJbase (16), to maximize the consistency between genotypes inferred using the ASC-based approach and information obtained by haplotype inference and genomic long-read data. Overall, 129 of 280 thresholds were adjusted. A list of all thresholds applied can be found in Supplementary Table S1.

The threshold listed in Supplementary Table S1 were determined from a cohort of northern European individuals. Hence, for cohorts with different ethnic backgrounds or from other species, adjustments to the allele threshold might be required. Therefore, to determine a new allele-based threshold for a different population, users should follow the following steps. First, the new AIRR-seq data should be aligned with the corresponding ASC germline reference set. Second, for each allele, the fraction of the allele usage from the total number of sequences should be derived. Third, if available, haplotype information should be extracted, and coupled with the allele usage from step two. Haplotype information can be obtained using an anchor gene such as IGHJ6, by separating the allele usage based on rearrangement with the anchor gene. Fourth, the default threshold should be either set to 10^−4^, or start from the determined allele-based threshold from Supplementary Table S1. Fifth, alleles should be grouped based on their ASCs, observing their usage against the starting allele-based threshold. The threshold should be adjusted in the following cases:

- **The allele is expressed at levels significantly higher than the default threshold in the study population**. In this case, the threshold needs to be increased, but remain below the lowest expression level amongst the studied individuals.
- **Haplotype evidence supports an allele expressed below the default threshold**. Here, we seek evidence that the allele is present on either one or both chromosomes, and if found, we adjust the threshold to include individuals expressing these alleles at low levels. However, in cases where we have specific information on the allele’s genomic location, such as with IUIS genes, it is necessary to ensure that there are no contradicting haplotype results. For instance, two alleles from the same IUIS gene should not appear on the same chromosome. If a contradiction is found, we maintain the current threshold until we gather more information on the possibility of gene duplication.
- **The study population expresses an undocumented allele**. If an undocumented allele was inferred to be present in the study population, first it should be attributed to the allele threshold of its closest allele, and then adjusted as in the two cases above.
  The cases we presented here are a guideline for determining the allele-based thresholds and adjusting from the default values.

### IGHV reference book

To further refine the threshold from specific populations of interest, we developed an interactive web server (https://yaarilab.github.io/IGHV_reference_book/) that presents the ASCs and the absolute frequencies of the alleles for datasets P1 and P11. Further, the server presents the chosen allele-specific thresholds shown in Supplementary Table S1. The server allows the end user to explore different choices for allele thresholds. This can be done using the interactive app on each ASC page, allowing the thresholds to be controlled by designated numeric input buttons. The app includes an interactive graph that displays the frequencies and thresholds. The interactive graph allows the user to explore haplotype information, if available, by ‘clicking’ the individual’s point on the graph. Once the user adjusts any of the thresholds, the app reloads with the adjusted threshold and presents the resulting genotypes. Modification of thresholds can be helpful in maximizing discrimination, particularly in cases where some alleles are have lower expression levels than others (15,24,26,27).

### AIRR-seq processing

To infer a personal genotype either by the ASC-based or the gene-based method (17), gene/allele assignments for each repertoire were determined using IgBLAST (V1.17) with the customized ASC germline reference set (DOI:10.5281/zenodo.7401239). Then, for each clone, a representative with the least number of mutations was chosen, undocumented alleles were inferred using TIgGER (17), and in cases for which new alleles were found, reassignment was carried out. If the dataset came from naive B cells, the sequences were filtered for no mutations within the V region up to position 316, accounting for possible sequencing errors at the end of the V region (24). For repertoires coming from full V region length amplicons, the repertoires were filtered to omit 5′ trimmed sequences. Sequences were also filtered based on whether there was sufficient 3′ coverage of the V region, requiring sequences to be at least 312 nucleotides long. For ASC-based inference, each allele’s absolute usage was calculated, and in cases in which a given sequence had more than a single assignment, the counts were divided among all clusters. For each allele within the repertoire, the absolute usage was compared to the specific threshold. Alleles that passed the threshold were then added to the final individual’s genotype. For the gene-based genotype inference, TIgGER’s ‘inferGenotype’ (17) function was used with the chosen threshold, either $12.5\%$ or $5\%$.

### Using genome long-read assemblies to validate alleles

Long-read assemblies from six samples generated using Sequel IIe HiFi reads and IGenotyper v1.0.0 (4) were used to validate the ASC-based genotype approach. The samples are those described previously (28,29). The assemblies were aligned to a custom immunoglobulin heavy chain (IGH) genome reference containing previously discovered IGHV genes using BLASR (4,30). From the alignments, fully phased gene sequences were extracted, including 5′ UTR, leader-1, leader-2 and exons. In 2 cases where a fully-phased gene sequence could not be extracted, the assembly was reviewed manually and a haplotype was determined on the basis of the coding region only. The matched repertoire sequences were processed as described in the ‘AIRR-seq processing’ subsection of the Methods. Because the sequences were pre-sorted to IGM, we inferred the genotype only for unmutated sequences (after inferring novel alleles). The comparison between the genomic annotation and the ASC-based genotype was made based on germline sequence similarity.

## RESULTS

### Allele naming system based on germline hierarchical structure

**Figure 2.**
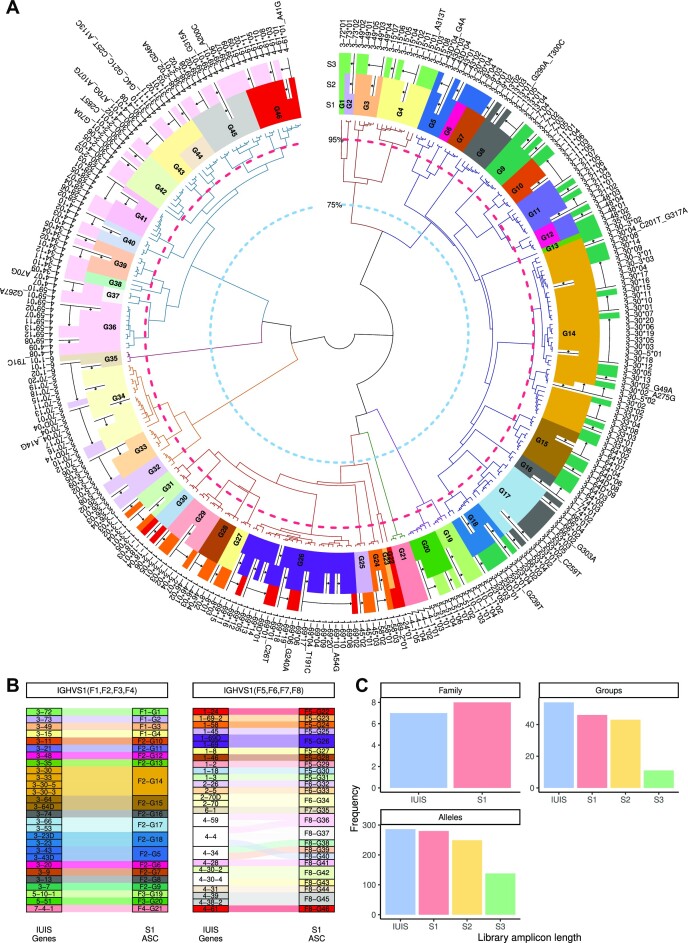
Allele Similarity Clusters. (**A**) Hierarchical clustering of the functional IGH germline reference set. The inner layer shows a dendrogram of the clustering. The dashed lines indicate the sequence similarity of $75\%$ (light blue) and $95\%$ (red). The dendrogram branches are colored by the $75\%$ sequence similarity. The inner colored circle shows the clusters and alleles for the library amplicon length of S1, the middle circle for the length of S2, and the outer for S3. The white spaces in each circle indicate alleles that cannot be distinguished within each respective germline reference set. The arrows indicate the direction and the extent of allele collapsing, with respect to the adjacent inner circle. (**B**) An alluvial plot showing the connection between the S1 ASC and the IUIS genes. The colors represent the S1 ASC. White represents IUIS genes whose alleles are clustered into more than a single allele similarity cluster. (**C**) The frequency of the subgroups/families, genes/clusters, and alleles for each amplicon length. The categories along the x-axis reflect the amplicon lengths and the y-axis is the count of the unique subgroups/families, genes/clusters, or alleles.

Using hierarchical clustering with complete linkage, we defined a two-level naming scheme for the set of functional germline alleles (downloaded from IMGT July 2022): allele families and ASCs. For the family level, we followed the logic and threshold of 75% nucleotide similarity from IMGT (31). Since we applied this methodology to the contemporary set of functional alleles, the resulting families mildly deviate from the IUIS family definitions. In particular, the IGHV3 subgroup is split into two families with our approach (Figure 2 A, the orange dashed circle defines the $75\%$ threshold). Using the same hierarchical tree, we clustered the sequences based on $95\%$ nucleotide similarity (Figure 2 A, blue dashed line). This resulted in 46 clusters, which we defined as ASCs, some of which consisted of several genes (Figure 2B). In addition, the alleles of some genes were split between different clusters. Adapting the two-level naming scheme resulted in an annotated germline reference set that reduced ambiguities in several analysis steps, as shown below. Moreover, as mentioned in the introduction, partial sequencing protocols exacerbate the computational challenges associated with the assignment of highly similar alleles originating from distinct genes. Our proposed naming scheme can be generalized in a straightforward way to these situations.

To adapt the above naming scheme to partial V sequences, we computationally trimmed the 5′ region of V sequences in the germline reference set according to sequence lengths obtained using the BIOMED-2 (13) and ImmunoSeq (14) protocols. For simplicity, we defined the sequencing protocols by the library amplicon length, and named the full-length amplicons ‘S1’, the partial V sequences corresponding to the BIOMED-2 style ‘S2’, and the minimal V coverage of ImmunoSeq ‘S3’ (Figure 2 A).

Depending on the amplicon length used, we obtained a different number of ASCs. As expected, the 5′ V trimming resulted in higher similarity between the alleles. Compared to the 54 genes in the IUIS database, after clustering we observed 46 ASCs in S1, 43 in S2 and 11 in S3 (Figure 2 C).

### ASC-based thresholds enhance genotyping accuracy

Many computational genotyping tools consider the relative frequency of a candidate allele during inference and filtering steps (17,22,32,33). An inference is made or accepted if the number of assignments to an allele exceeds a threshold percentage of the total assignments to all alleles of the corresponding gene. A need for allele-specific filtering processes has been raised previously (27). Here, we implemented a method based on the explicit comparison of each allele’s frequency in the repertoire under study with an allele-specific threshold. In the presented method, an allele enters the genotype if its absolute frequency exceeds its allele-specific threshold. The genotype inference process was done using the ASC naming scheme, such that identical nucleotide sequences from different chromosomal positions were collapsed into a single allele annotation. This addressed issues that can confound frequency observations in current methods: variable expression levels between alleles of the same gene, multiple assignments, duplicated genes, and short reads.

**Figure 3.**
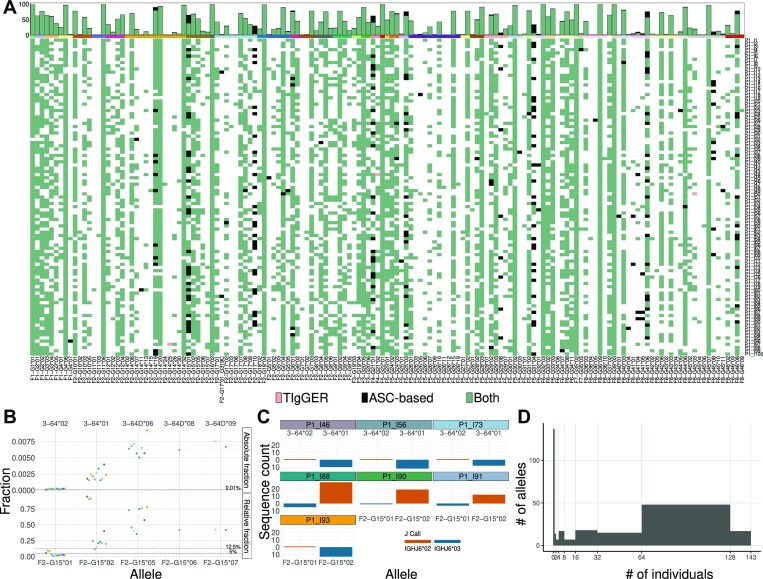
Genotype inference comparison between the ASC-based and TIgGER fraction method. (**A**) A heatmap comparing the genotypes inferred from TIgGER (17) and from the ASC-based method. The bottom panel is the heatmap comparison, where each row is a genotype inference of an individual from the P1 dataset and each column is a different allele. The alleles shown in the heatmap are those for whom at least one of the methods inferred a genotype. Black and Pink colors represent alleles that only entered the genotype either in the ASC-based method or in TIgGER (17) with a $12.5\%$ threshold, respectively. Green represents alleles that entered the genotype in both methods, and white represents alleles that did not pass in both methods. The top panel is the summation of the heatmap events. The y-axis is the count of the individuals for whom a given allele entered the genotype. The x-axis is the different alleles. The colored strip bar shows the alleles ASC group and matches the colors of those shown in Figure 2(B) The relative and absolute frequencies of the ASC IGHVF2-G15. Each dot is an individual for whom allele 01 entered the genotype with the ASC-based method, but did not in TIgGER (17). The colors represent the different individuals. Each column is a different allele from the cluster. The top row is the absolute frequency and the bottom is for the relative frequency. (**C**) Haplotype inference based on IGHJ6 for the individuals from (**B**). Each facet is a different individual, and the facet color matches the dots from (B). In each facet, the top row and orange color is the frequency for the IGHJ6*02 chromosome and the bottom and blue color for the IGHJ6*03 chromosome. The x-axis is the different alleles for the cluster, and the y-axis is the sequence count. (**D**) A histogram of the allele abundance distribution in the studied population. The x-axis is the number of individuals attributed to each allele, and the y-axis is the number of alleles.

We started with a default value for the allele-specific threshold (10^−4^), and assessed it allele by allele by comparing the outcome of the genotype for this value with the picture that emerges from a complementary haplotype inference (see methods). After reviewing and, where necessary, adjusting the allele-specific thresholds for all the IGHV alleles observed in two naive cell datasets, VDJbase projects P1 (24) and P11 (PRJEB58016), 142 repertoires in total, we compared the resulting inferred genotypes with the ones inferred by TIgGER, which is a gene-based inference tool (Figure 3 A for P1, and Supplementary Figure S3 for P11). In the ASC-based genotype approach, alleles enter the genotype if their usage is higher than the allele-specific thresholds. In TIgGER’s genotype inference, on the other hand, the alleles enter the genotype based on the relative usage normalized by all sequences mapped to this gene. A common step in this kind of analysis includes an undocumented allele inference. In the ASC-based genotype inference, each inferred undocumented allele is given the allele-specific threshold of its most similar allele. Overall, there were 5767 allele calls that were included in either one or both genotypes. Results were concordant for inference of highly used alleles, with 5548 allele calls that fully matched between the methods ($∼96\%$, green squares). However, there were 3 allele calls that only entered the genotype with TIgGER (pink squares), and 216 alleles that were called only by the ASC-method (black squares).

The potential false positives in genotypes inferred by TIgGER (17) were seen in cases where all observed alleles of a particular gene were expressed at low levels, according to population data. An example is IGHV7-4-1 (part of ASC IGHVS1F4-G21). In all individual genotype inferences, there was not a single situation of heterozygosity for this cluster, as in most individuals there was one dominant allele. In the single occasion where heterozygosity was declared, both alleles *01 and *02 entered the genotype. However, the inference of allele *02 is likely to be incorrect. In this particular sample (VDJbase: P1_I44_S1), the poorly expressed allele was *01, with 4 sequences, while the highly expressed allele *02 only had a single sequence. This deviates from what is seen in the population. The three alleles attributed to this cluster vary in usage, with allele *02 being the most expressed allele (27) with a median usage of 1.19 × 10^−2^. This is 33 times more than the second expressed allele (*01). Hence, the situation where allele *01 dominates over allele *02 is unlikely (*P* value of 4 × 10^−6^ according to a binomial test), and the identification of a read associated to allele *02 might be the result of a mutation or a PCR or sequencing error. This indicates clear deviations between the approaches that may lead to different specificities in clusters with low expression.

Potential false negatives in the genotypes inferred by TIgGER (17) are seen in cases where one allele is expressed at a lower rate than other alleles of the gene, according to population data. An example is IGHV3-64*02, corresponding to ASC IGHVS1F2-G15*01. This allele entered the genotype using the ASC-based method, but not using the conventional TIgGER methodology (17). The IGHVS1F2-G15 cluster combines alleles from two IUIS genes, IGHV3-64 and IGHV3-64D, which merge under the $95\%$ threshold. The alleles of this cluster vary in usage, i.e., alleles *05, *06, and *07, typically assigned to IGHV3-64D, are more frequently used than *02 and *01 (Figure 3 B). Allele IGHVS1F2-G15*01 (IGHV3-64*02) is expressed at a considerably lower level than the other alleles, with a median of 2.1 × 10^−4^ absolute frequency: roughly 12 times lower than the second most lowly expressed allele, IGHVS1F2-G15*02 (aka IGHV3-64*01). Despite the fact that allele IGHVS1F2-G15*01 was above the ASC-based default threshold (10^−4^) and as such entered the genotype, it is far below TIgGER’s (17) relative fraction threshold of $12.5\%$ or $5\%$, as it has a median relative frequency of $1.86\%$ (Figure 3 B). This raised the question of whether the alleles with low expression truly exist. To validate the inference of allele IGHVS1F2-G15*01, we looked at the haplotype of alleles IGHVS1F2-G15*01 and IGHVS1F2-G15*02, since they come from the same chromosomal location (IGHV3-64). We haplotyped seven individuals who ostensibly included allele *01 with the ASC-method but not in TIgGER (17), using heterozygosity at IGHJ6 (15) as the anchor (Figure 3 C). In all seven individuals, alleles *01 and *02 were found on opposite chromosomes, strongly supporting the presence of allele IGHVS1F2-G15*01. This example demonstrated the sensitivity of the ASC-based approach to inferences of alleles with low expression, which may provide important insights for future studies.

Figure 3 D summarizes the distribution of allele prevalence in cohorts P1 and P11. Seven out of the 280 alleles present in the ASC germline reference set appeared in all 142 individuals, while $41\%$ of the alleles, 116 out of 280, did not enter any of the genotypes. This implies that reference sets should potentially be population-specific (34,35), or that the current reference set includes a large fraction of unexpressed or non-existent alleles (36).

### Allele usage reporting

**Figure 4.**
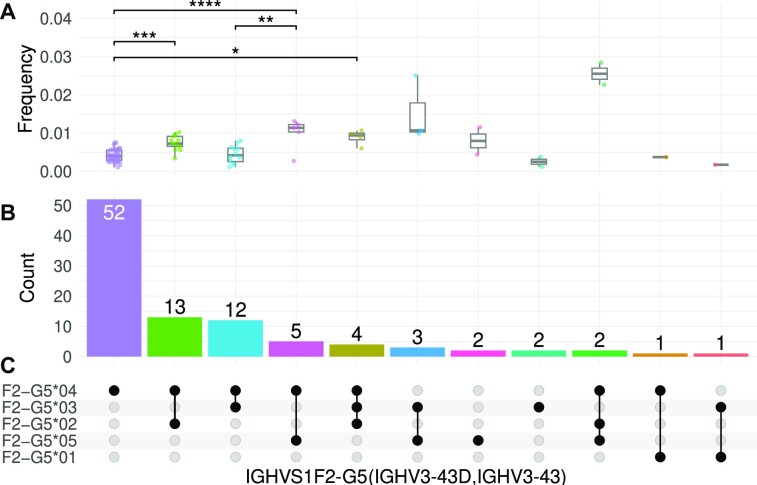
Gene usage is associated with genotype. (**A**) The y-axis is the genotype allele combination frequencies of IGHVS1F2-G5, normalized by the number of sequences in the whole repertoire, for individuals in P1. The x-axis is the genotype allele combination, ordered by the number of individuals carrying the combinations (**B**). Each point is an individual, and colors represent the order of the genotype allele combination. A Tukey’s HSD multiple comparison test was performed for groups containing four individuals or more. The adjusted p values are indicated on the connecting line; only the statistically significant combinations were drawn. ns: *P* > 0.05, *P ≤ 0.05, **P ≤ 0.01, ***P ≤ 0.001, ****P ≤ 0.0001. (**C**) The genotype allele combination intersect matrix. Each row is a different allele and each column is a different genotype allele combination.

Subgroups, genes, and sometimes alleles are commonly used as AIRR-seq features, for example in reporting over-expression of specific genes or families in the context of specific diseases. These features are highly sensitive to the nomenclature and the genotypes of the individuals in the cohort. Here, we compared the reporting of allele-level usage versus gene or cluster level. Supplementary Figure S4 shows that reporting of usage was highly influenced by the genotype of the individuals. Figure 4 shows a more in depth comparison for cluster IGHVS1F2-G5, for which the observed mean absolute usage in individuals who carry alleles *04 and *05 was significantly higher than in those who carry *03 and *04 (Figure 4A). If we had presented the usage results in an aggregated manner, however, disregarding the genotype allele combination attribute, this variation would have been masked (Supplementary Figure S4, left upper panel).

Moreover, if IUIS gene names were used to report the usage of these alleles, it would have been split between the V3-43 and V3-43D columns. Consequently, when studying allele usage in human cohorts, we recommend that usage is reported at the allele or ASC levels, to avoid unnecessary ambiguities.

### Genomic validation of the ASC-based genotype

**Figure 5.**
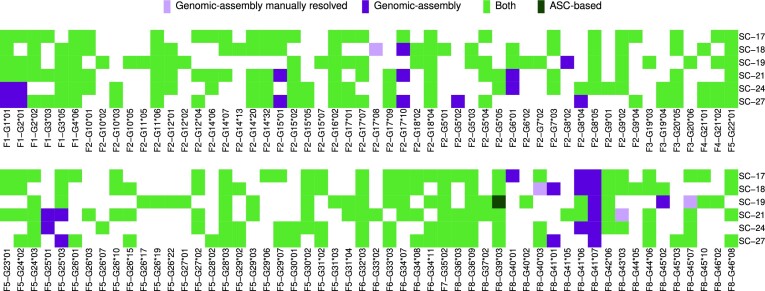
Genomic inference versus AIRR-seq genotype inference. A heatmap of a comparison between the ASC-based genotype method and genomic validation. Each row is an individual, and each column is a different allele. The alleles shown in the heatmap are those for which at least one of the methods inferred a genotype. Dark green and dark purple represent alleles that are only present in the ASC-based method or genomic validation, respectively. Light green represents alleles that are present in both methods, and white represents alleles that are not seen in either. Light purple represents alleles of genes that were resolved with manual inspection.

We validated our ASC-based genotyping method using a paired dataset drawn from six subjects, comprising full length AIRR-seq repertoire sequencing of IGM from RNA isolated from PBMCs, and haplotype-partitioned assemblies of the genomic IGHV locus derived from long-read sequencing (29). Across the six subjects (Figure 5), a total of 304 ASC allele calls were made from the AIRR-seq repertoires, counting the inference of a single allele in a single individual as an allele call. The comparison between the ASC allele calls with the genomic annotation was made based on sequence similarity. In three subjects, genomic assemblies were not fully resolved using IGenotyper, either not spanning certain genes, or diploid assemblies representing both haplotypes were not resolved for all genes. This resulted in an inability to validate 4 of the 304 inferences made from the AIRR-seq analysis. However, with manual examination of the assemblies we were able to validate them (Figure 5, light purple). For the remaining 300 inferred allele calls, 299 were concordant between the ASC and genomic results (light green squares). Meaning that overall we were able to match 303 of the calls with the genomic assemblies (>99.6 percent). The single discordant allele was IGHVS1F8-G39*03 (IGHV4-34*01) in subject SC-19 (dark green square); in this case, an undocumented IGHV4-34 allele was annotated from the genomic data, characterized by novel SNPs located at the very 3′ end of the V region (position 319, and 320). The 3′ end of the V region is often trimmed upon recombination, hence, as mentioned above, inferring an undocumented allele from AIRR-seq repertoire sequences based on these positions is unreliable (7). A peculiar example involved the inferred allele IGHVS1F8-G36*03 (IGHV4-59*08). This allele has been speculated to reside at gene IGHV4-61 (27,37,38). Directly supporting this, our comparison showed that the germline sequence of the allele perfectly matched the long-read assemblies spanning IGHV4-61. Thirty-one allele calls were found only in the genomic samples (dark purple squares) and not in the ASC genotypes, implying that these alleles are poorly or not at all expressed. Such examples have been described in the literature (24,27,39).

In summary, out of the 304 allele calls that were made across six individual genotypes using the ASC-based method, we found potential contradictions from the genomic data only in two cases. These cases most likely indicate technical issues with the genomic assembly due to reduced coverage, rather than in the ASC-based genotype inference method.

### Generalizability to other germline reference sets

**Figure 6.**
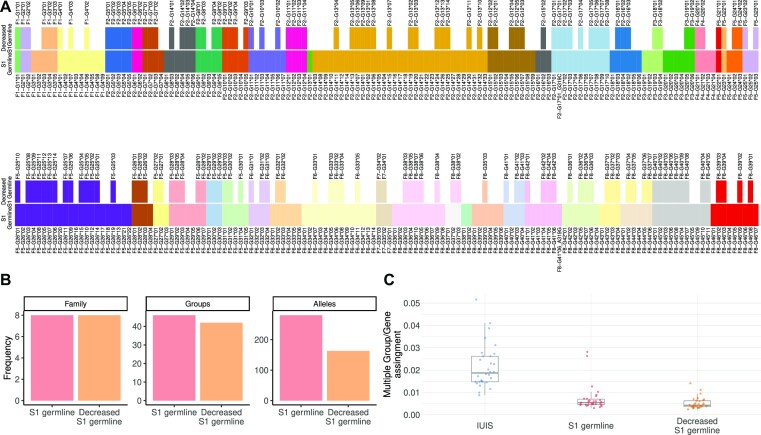
The effects of an incomplete germline reference set. (**A**) The heatmap shows the clusters based on the full germline used in Figure 2, and the re-clustering after the reduced germline, which includes alleles that entered the genotype using the ASC-based method on the P1 and P11 cohorts. The bottom row shows the clusters for the full germline reference set, and the top row shows the clusters for the new germline. The colors represent the different clusters. White represents alleles that did not enter the genotype. (**B**) Summation of the number of families, clusters, and alleles in the S1 germline and the reduced reference. The x-axis is the different reference sets and the y-axis is the count of the events. (**C**) The frequency of multiple cluster/gene assignments. The x-axis is the different reference sets and the y-axis is the absolute frequency of multiple assignments. Each dot is an individual’s multiple assignment frequency from the non-naive P4 cohort.

One potential limitation of the proposed naming scheme is that the specific alleles in the germline reference set determine the allele families and ASCs, hence the clustering may change when alleles are added or removed from the set. To quantify the impact of an altered germline reference set, we created a reduced germline reference set consisting only of the alleles that entered the genotype of P1 individuals, as determined by our ASC-based method. This is an example of transferring one germline reference set from one dataset to another without adjusting it. We then applied the clustering algorithm and obtained the new families and clusters (Figure 6 A). Compared to the original set, two cluster pairs were merged, G36/G37 and G43/G44, and G13 and G38 were dropped, as none of their alleles entered any of the genotypes. As shown in (Figure 6 A), the overall structure of the clusters was maintained except the relatively minor changes detailed above, despite the drastic reduction from 280 to 164 alleles (Figure 6 B). From this, we conclude that the clustering method is relatively robust to changes in the reference set composition.

To further assess the flexibility and effect of the reference set, we tested the multiple assignments in a non-naive repertoire. Multiple assignments are cases in which the aligner, IgBLAST in our evaluations, cannot determine a single matched allele, and outputs multiple options for the most likely germline allele. This can be caused by sequencing errors, somatic hypermutation, identical germline alleles shared by multiple genes, or a combination. We explored this effect using the P4 dataset from VDJbase, which included non-naive repertoires from 28 individuals. We aligned the repertoires three times, once with the IUIS gene definitions downloaded from IMGT (the IMGT set), once with an identical set of sequences but using the proposed assignment nomenclature (the S1 set), and once with the reduced germline reference set described above (the reduced S1 set). We calculated the fraction of sequences that were attributed by the aligner to more than a single gene/ASC. Figure 6C shows an expected, significant 3-fold reduction in multiple assignments between the IMGT set and the S1 set. The reduced S1 set showed a further reduction in multiple assignments.

**Figure 7.**
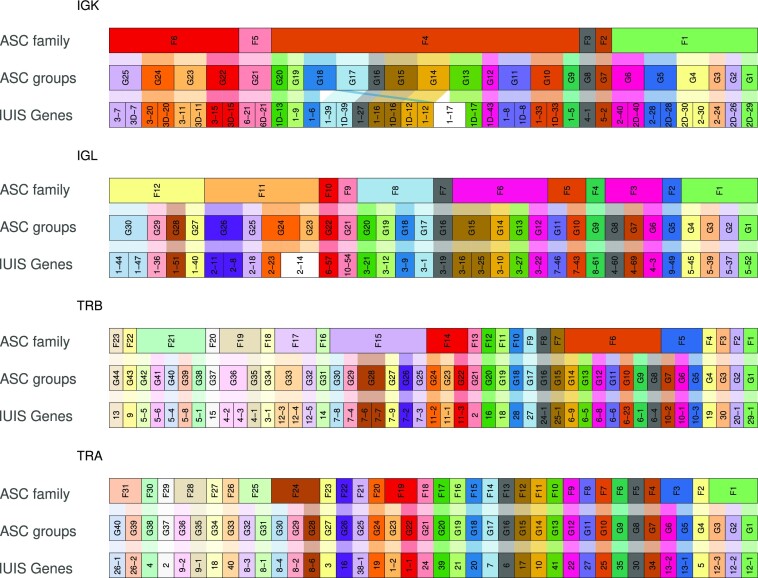
Allele clusters for V genes from IGL/K and TRB/A loci. Each alluvial plot represents the cluster division for a given locus. The first row of each plot shows the division of the families, the second row the ASCs, and the third IUIS gene clustering. The colors represent the allele clusters. White represents IUIS genes that have been re-clustered into more than a single allele cluster.

We then applied the ASC approach to other AIR-encoding genomic loci. We clustered the sets of functional alleles downloaded from IMGT (July 2022) for human IGKV, IGLV, TRBV, and TRAV. We applied the same thresholds of 75% and 95% for determining the allele families and ASCs (Figure 7). The IGK locus is unique because it harbors two large duplicated V gene blocks that are inversely oriented, separated by a large region enriched with complex repeats. Here, as in IGHV, some genes share alleles with identical sequences. As expected, these duplicated alleles are clustered together under the 95% threshold. A split is observed in IGKV1-17, whose alleles are assigned to two ASCs. In the IGL locus, where the IUIS nomenclature defines 10 subgroups, we found 12 families using our approach and thresholds. Four genes were combined into ASCs, and a single gene was split into two ASCs. The loci of TRB and TRA remained relatively constant, except for four TRBV genes, which were merged into two ASCs. We developed an interactive application that applies the ASC naming scheme to V allele reference sets from different loci and organisms, https://yaarilab.github.io/IGHV_reference_book/alleles_groups.html. As the reference set can change over time, we recommend not using the nomenclature in reporting but only in the downstream analyses. Nevertheless, for backtracking, reproducibility, and interoperability, we maintain a Zenodo archive (https://doi.org/10.5281/zenodo.7401189) of all ASC runs conducted by our web server. It allows translation of the allele cluster names into IUIS names and also into the unique names suggested in the supplementary materials (Supplementary Table S1).

## DISCUSSION

Many studies have used repertoire sequencing data to explore the IG and TR loci using inference tools (17,32,33,40–43). As a consequence, a plethora of allele sequences have been discovered (7–10,24,44–47). In many cases, alleles can be assigned to specific genes. However, in some cases, gene duplications and extensive inter-individual haplotype variations (including structural variants yet to be identified) present challenges for the the accurate assignment of alleles to genes (27). In less documented species, genomic loci are often not extensively characterized (48). For example, rhesus macaque and mouse germline reference sets have been published as discrete sets of allele sequences without gene attributions (45,49).

In this study, we report on two innovations that can be highly beneficial in such situations. The first is our proposed naming scheme that organizes alleles into clusters based on sequence similarity, which aids downstream analyses. The ASCs can be used for clonal inference, usage reporting, and genotype and haplotype inferences. We propose that the ASC naming scheme can be utilized for AIRR-seq analysis in understudied species until comprehensive characterizations of genomic loci are conducted. That being said, the proposed scheme is not meant to replace the existing IUIS naming, but rather to accompany it and allow for a more inclusive analysis. We created an R package (PIgLET, https://bitbucket.org/yaarilab/piglet), which includes an example dataset for testing purposes, and an online application within the ASC website (https://yaarilab.github.io/IGHV_reference_book/alleles_groups.html). This tool allows users to infer ASCs based on their own IGHV allele reference set and plot the ASC results. For better results, we recommend using the ASC germline reference set as a personalized database for aligner softwares such as IgBLAST. Further, we suggest the user to archive its ASC germline reference set using a Zenodo doi. We created an archive in Zenodo (10.5281/zenodo.7401239) for the ASC germline reference set presented here. The archive will be updated as we release new reference sets. As mentioned, a potential use case for our proposed naming scheme is for clonal inference methods. Many of the clonal inference methods rely on V gene assignments, which are impacted by similar genes and alleles. Therefore, utilizing the ASC approach may lead to better clonal inference performance.

The second innovation we report is a new and improved approach for inference of a personal genotype and for determination of VDJ allele usage from AIRR-seq data. The approach is based on the *absolute* frequency of allele usage within a specific population, rather than on *relative* usage , which is normalized at the gene level. We created an interactive website where each page shows the allele usage across the naive IGHV repertoires from P1 and P11 studies of VDJbase. The site allows users to view the default ASC-based allele-specific thresholds, and to explore the implications of changing these values. Altering the default values is primarily advised for experienced users who wish to adapt these values for different populations or species. Our site will be continuously updated as more naive AIRR-seq and direct genomic sequencing datasets accumulate. Along with the site, the thresholds for allele detection in VDJbase will also be updated. Moreover, as new species are sequenced and published, we will include them in the site and update VDJbase accordingly.

In summary, the ASC approach creates a clean dataset for analytics. It transforms an existing germline reference set into a set of sequences that correctly represent the assignments that can be made in a repertoire. This is analogous to the data preparation step in machine learning. When analysis is complete, results can be transformed back into the language of the existing data set, at which point the ambiguities imposed by the limitations of the experiment are made clear. As an example, consider an IGH repertoire derived from reads amplified with BIOMED-2 primers. Using the S2 germline reference set provided with the ReferCreated with BioRender.comence Book, analysis might establish that allele IGHVF2-G10*09 is expressed at a high level compared to baseline. Translating back to IUIS nomenclature, the equivalent result is that ‘a combination of alleles IGHV3-53*03 and IGHV3-53*02 are expressed at a high level relative to baseline’. It is not possible to tell from the repertoire whether the over-expression is related to allele *03 or *02, because they are indistinguishable in the reads, as the primers used mask differences between them. The ASC terminology correctly addresses this, without the need to manage the ambiguity explicitly in the analytics pipeline. Users can also apply the approach in circumstances where there is no existing gene-based germline reference set, by using the capabilities provided in PIgLET. This will create a gene-like reference set, and a baseline expression level for each allele. Because PIgLET’s personalized genotyping is based on the expression levels of individual alleles, it can be used on repertoires prepared in this way, and hence improve the quality of assignments.

We have demonstrated the application of ASC-based allele usage information to an allele expression analysis in AIRR-seq studies (Figure 4), and show how it reduces the number of multiple gene assignments (Figure 6). The resulting allele usage vector provides a clear signal, tailored to the details of the underlying data set, which can be used in graphical reports or machine learning applications. The conclusions can be translated back to IUIS nomenclature.

It is known that some alleles of a gene may be expressed at higher levels than other alleles. An important feature of the ASC-based genotype method, is its ability to accurately assess the validity of alleles with low expression. Identifying such alleles with low expression and including them in the genotype can be critical for investigating disease susceptibility (39,43,50–54).

We validated the ASC-based approach by comparing AIRR-seq inferred genotypes with a genotype based on direct long read genomic sequencing (4). Even though some repertoires in these genomically sequenced cohorts had relatively low AIRR-seq depths, the comparison showed a strong concordance between the direct sequencing and the proposed inference method.

The set of unique alleles inferred from two large studies in VDJbase by our method constitutes less than 60% of the current IGHV reference available in IMGT. This brings up again the interesting debate of whether all alleles in the existing reference set truly exist. This point was previously reviewed in (36), in which the authors discovered that several alleles were erroneous. On the other hand, many ethnic populations are understudied (35), and most likely store many more alleles to be discovered. We believe that these matters should be further discussed and reviewed to curate an optimal reference set for AIRR-seq analyses. As demonstrated by Rodriguez *et al.* (28) different ethnic backgrounds influence the IGH composition (i.e., genes, deletions, etc.). Our study is based on samples solely derived from a northern European setting. With more repertoire data curated from individuals with different ethnic backgrounds, the allele-specific threshold might have to be tailored to the different populations. We envisage that with the rising interest in AIRR-seq, future studies will reveal more diversity, which will contribute to the efforts to enhance both the ASC website and VDJbase, and to the optimization of inferences and tools.

## Supplementary Material

gkad603_Supplemental_File

## Data Availability

The IGH BCR repertoires sequences for P1 (PRJEB26509), P4, and P11 (PRJEB58016) are available on European Nucleotide Archive. The figure and the supporting tables for creating them are available in the GitHub repository (https://github.com/yaarilab/IGHV_ASC_manuscript, DOI:10.5281/zenodo.8018867), and the ASCs clusters are available in the GitHub repository (https://github.com/yaarilab/asc_archive, DOI:10.5281/zenodo.7401239). The interactive website (https://yaarilab.github.io/IGHV_reference_book/) is available in the GitHub repository (https://github.com/yaarilab/IGHV_reference_book, DOI:10.5281/zenodo.7513021). The PIgLET R package is available at the Bitbucket repository https://bitbucket.org/yaarilab/piglet and the documentation is available at https://piglet.readthedocs.io.

## References

[1] Trück J. , Eugster A., Barennes P., Tipton C.M., Luning Prak E.T., Bagnara D., Soto C., Sherkow J.S., Payne A.S., Lefranc M.-P. et al. Biological controls for standardization and interpretation of adaptive immune receptor repertoire profiling. Elife. 2021; 10:e66274. 34037521 10.7554/eLife.66274 PMC8154019

[2] Matsuda F. , Ishii K., Bourvagnet P., Kuma K.-I., Hayashida H., Miyata T., Honjo T. The complete nucleotide sequence of the human immunoglobulin heavy chain variable region locus. J. Exp. Med. 1998; 188:2151–2162. 9841928 10.1084/jem.188.11.2151 PMC2212390

[3] Watson C.T. , Steinberg K.M., Huddleston J., Warren R.L., Malig M., Schein J., Willsey A.J., Joy J.B., Scott J.K., Graves T.A., et al. Complete haplotype sequence of the human immunoglobulin heavy-chain variable, diversity, and joining genes and characterization of allelic and copy-number variation. Am. J. Hum. Genet. 2013; 92:530–546. 23541343 10.1016/j.ajhg.2013.03.004 PMC3617388

[4] Rodriguez O.L. , Gibson W.S., Parks T., Emery M., Powell J., Strahl M., Deikus G., Auckland K., Eichler E.E., Marasco W.A. et al. A novel framework for characterizing genomic haplotype diversity in the human immunoglobulin heavy chain locus. Front. Immunol. 2020; 11:2136. 33072076 10.3389/fimmu.2020.02136 PMC7539625

[5] Kodaira M. , Kinashi T., Umemura I., Matsuda F., Noma T., Ono Y., Honjo T. Organization and evolution of variable region genes of the human immunoglobulin heavy chain. J. Mol. Biol. 1986; 190:529–541. 3097326 10.1016/0022-2836(86)90239-1

[6] Giudicelli V. , Lefranc M.-P. Ontology for immunogenetics: the IMGT-Ontology. Bioinformatics. 1999; 15:1047–1054. 10745995 10.1093/bioinformatics/15.12.1047

[7] Mikocziova I. , Peres A., Gidoni M., Greiff V., Yaari G., Sollid L.M. Germline polymorphisms and alternative splicing of human immunoglobulin light chain genes. Iscience. 2021; 24:103192. 34693229 10.1016/j.isci.2021.103192 PMC8517844

[8] Mikocziova I. , Gidoni M., Lindeman I., Peres A., Snir O., Yaari G., Sollid L.M. Polymorphisms in human immunoglobulin heavy chain variable genes and their upstream regions. Nucleic Acids Res. 2020; 48:5499–5510. 32365177 10.1093/nar/gkaa310 PMC7261178

[9] Omer A. , Peres A., Rodriguez O.L., Watson C.T., Lees W., Polak P., Collins A.M., Yaari G. T cell receptor beta germline variability is revealed by inference from repertoire data. Genome Med. 2022; 14:2. 34991709 10.1186/s13073-021-01008-4 PMC8740489

[10] Vázquez Bernat N. , Corcoran M., Hardt U., Kaduk M., Phad G.E., Martin M., Karlsson Hedestam G.B. High-quality library preparation for NGS-based immunoglobulin germline gene inference and repertoire expression analysis. Front. Immunol. 2019; 10:660. 31024532 10.3389/fimmu.2019.00660 PMC6459949

[11] Gibson W.S. , Rodriguez O.L., Shields K., Silver C.A., Dorgham A., Emery M., Deikus G., Sebra R., Eichler E.E., Bashir A. et al. Characterization of the immunoglobulin lambda chain locus from diverse populations reveals extensive genetic variation. Genes Immun. 2023; 24:21–31. 36539592 10.1038/s41435-022-00188-2 PMC10041605

[12] Zhang B. , Meng W., Prak E. T.L., Hershberg U. Discrimination of germline V genes at different sequencing lengths and mutational burdens: a new tool for identifying and evaluating the reliability of V gene assignment. J. Immunol. Methods. 2015; 427:105–116. 26529062 10.1016/j.jim.2015.10.009 PMC4811607

[13] van Dongen J.J.M. , Langerak A.W., Brüggemann M., Evans P.A.S., Hummel M., Lavender F.L., Delabesse E., Davi F., Schuuring E., García-Sanz R. et al. Design and standardization of PCR primers and protocols for detection of clonal immunoglobulin and T-cell receptor gene recombinations in suspect lymphoproliferations: Report of the BIOMED-2 Concerted Action BMH4-CT98-3936. Leukemia. 2003; 17:2257–2317. 14671650 10.1038/sj.leu.2403202

[14] Morin A. , Kwan T., Ge B., Letourneau L., Ban M., Tandre K., Caron M., Sandling J.K., Carlsson J., Bourque G. et al. Immunoseq: the identification of functionally relevant variants through targeted capture and sequencing of active regulatory regions in human immune cells. BMC Med. Genom. 2016; 9:59. 10.1186/s12920-016-0220-7 PMC5022205 27624058

[15] Peres A. , Gidoni M., Polak P., Yaari G. RAbHIT: R antibody haplotype inference tool. Bioinformatics. 2019; 35:4840–4842. 31173062 10.1093/bioinformatics/btz481

[16] Omer A. , Shemesh O., Peres A., Polak P., Shepherd A.J., Watson C.T., Boyd S.D., Collins A.M., Lees W., Yaari G. VDJbase: an adaptive immune receptor genotype and haplotype database. Nucleic Acids Res. 2020; 48:D1051–D1056. 31602484 10.1093/nar/gkz872 PMC6943044

[17] Gadala-Maria D. , Yaari G., Uduman M., Kleinstein S.H. Automated analysis of high-throughput B-cell sequencing data reveals a high frequency of novel immunoglobulin V gene segment alleles. Proc. Natl. Acad. Sci. U.S.A. 2015; 112:E862–E870. 25675496 10.1073/pnas.1417683112 PMC4345584

[18] Ralph D.K. , Matsen IV F.A. Per-sample immunoglobulin germline inference from B cell receptor deep sequencing data. PLoS Comput. Biol. 2019; 15:e1007133. 31329576 10.1371/journal.pcbi.1007133 PMC6675132

[19] Rosenfeld A.M. , Meng W., Luning Prak E.T., Hershberg U. ImmuneDB, a novel tool for the analysis, storage, and dissemination of immune repertoire sequencing data. Front. Immunol. 2018; 9:2107. 30298069 10.3389/fimmu.2018.02107 PMC6161679

[20] Yaari G. , Kleinstein S.H. Practical guidelines for B-cell receptor repertoire sequencing analysis. Genome Med. 2015; 7:121. 26589402 10.1186/s13073-015-0243-2 PMC4654805

[21] Gupta N.T. , Adams K.D., Briggs A.W., Timberlake S.C., Vigneault F., Kleinstein S.H. Hierarchical clustering can identify B cell clones with high confidence in Ig repertoire sequencing data. J. Immunol. 2017; 198:2489–2499. 28179494 10.4049/jimmunol.1601850 PMC5340603

[22] Slabodkin A. , Chernigovskaya M., Mikocziova I., Akbar R., Scheffer L., Pavlović M., Bashour H., Snapkov I., Mehta B.B., Weber C.R., et al. Individualized VDJ recombination predisposes the available Ig sequence space. Genome Res. 2021; 31:2209–2224. 34815307 10.1101/gr.275373.121 PMC8647828

[23] Gadala-Maria D. , Gidoni M., Marquez S., Vander Heiden J.A., Kos J.T., Watson C.T., O’Connor K.C., Yaari G., Kleinstein S.H. Identification of subject-specific immunoglobulin alleles from expressed repertoire sequencing data. Front. Immunol. 2019; 10:129. 30814994 10.3389/fimmu.2019.00129 PMC6381938

[24] Gidoni M. , Snir O., Peres A., Polak P., Lindeman I., Mikocziova I., Sarna V.K., Lundin K.E., Clouser C., Vigneault F. et al. Mosaic deletion patterns of the human antibody heavy chain gene locus shown by Bayesian haplotyping. Nat. Commun. 2019; 10:628. 30733445 10.1038/s41467-019-08489-3 PMC6367474

[25] Eliyahu S. , Sharabi O., Elmedvi S., Timor R., Davidovich A., Vigneault F., Clouser C., Hope R., Nimer A., Braun M. et al. Antibody repertoire analysis of Hepatitis C virus infections identifies immune signatures associated with spontaneous clearance. Front. Immunol. 2018; 9:3004. 30622532 10.3389/fimmu.2018.03004 PMC6308210

[26] Kidd M.J. , Chen Z., Wang Y., Jackson K.J., Zhang L., Boyd S.D., Fire A.Z., Tanaka M.M., Gaëta B.A., Collins A.M. The inference of phased haplotypes for the immunoglobulin H chain V region gene loci by analysis of VDJ gene rearrangements. J. Immunol. 2012; 188:1333–1340. 22205028 10.4049/jimmunol.1102097 PMC4734744

[27] Ohlin M. Poorly expressed alleles of several human immunoglobulin heavy chain variable genes are common in the human population. Front. Immunol. 2021; 11:603980. 33717051 10.3389/fimmu.2020.603980 PMC7943739

[28] Rodriguez O.L. , Safonova Y., Silver C.A., Shields K., Gibson W.S., Kos J.T., Tieri D., Ke H., Jackson K.J., Boyd S.D. et al. Genetic variation in the immunoglobulin heavy chain locus shapes the human antibody repertoire. 2022; bioRxiv doi:11 September 2022, preprint: not peer reviewed 10.1101/2022.07.04.498729. PMC10362067 37479682

[29] Ford E.E. , Tieri D., Rodriguez O.L., Francoeur N.J., Soto J., Kos J.T., Peres A., Gibson W.S., Silver C.A., Deikus G. et al. FLAIRR-Seq: a method for single-molecule resolution of near full-length antibody H chain repertoires. J. Immunol. 2023; 210:1607–1619. 37027017 10.4049/jimmunol.2200825 PMC10152037

[30] Chaisson M.J. , Tesler G. Mapping single molecule sequencing reads using basic local alignment with successive refinement (BLASR): application and theory. BMC Bioinformatics. 2012; 13:238. 22988817 10.1186/1471-2105-13-238 PMC3572422

[31] Lefranc M.-P , Lefranc G The immunoglobulin factsbook. 2001; Academic press.

[32] Gadala-Maria D. , Gidoni M., Marquez S., Vander Heiden J.A., Kos J.T., Watson C.T., O’Connor K.C., Yaari G., Kleinstein S.H. Identification of subject-specific immunoglobulin alleles from expressed repertoire sequencing data. Front. Immunol. 2019; 10:129. 30814994 10.3389/fimmu.2019.00129 PMC6381938

[33] Corcoran M.M. , Phad G.E., Bernat N.V., Stahl-Hennig C., Sumida N., Persson M.A., Martin M., Hedestam G.B.K. Production of individualized V gene databases reveals high levels of immunoglobulin genetic diversity. Nat. Commun. 2016; 7:13642. 27995928 10.1038/ncomms13642 PMC5187446

[34] Wang Y. , Jackson K.J., Gäeta B., Pomat W., Siba P., Sewell W.A., Collins A.M. Genomic screening by 454 pyrosequencing identifies a new human IGHV gene and sixteen other new IGHV allelic variants. Immunogenetics. 2011; 63:259–265. 21249354 10.1007/s00251-010-0510-8

[35] Peng K. , Safonova Y., Shugay M., Popejoy A.B., Rodriguez O.L., Breden F., Brodin P., Burkhardt A.M., Bustamante C., Cao-Lormeau V.-M. et al. Diversity in immunogenomics: the value and the challenge. Nat. Methods. 2021; 18:588–591. 34002093 10.1038/s41592-021-01169-5 PMC8842483

[36] Wang Y. , Jackson K.J., Sewell W.A., Collins A.M. Many human immunoglobulin heavy-chain IGHV gene polymorphisms have been reported in error. Immunol. Cell Biol. 2008; 86:111–115. 18040280 10.1038/sj.icb.7100144

[37] Parks T. , Mirabel M.M., Kado J., Auckland K., Nowak J., Rautanen A., Mentzer A.J., Marijon E., Jouven X., Perman M.L. et al. Association between a common immunoglobulin heavy chain allele and rheumatic heart disease risk in Oceania. Nat. Commun. 2017; 8:14946. 28492228 10.1038/ncomms14946 PMC5437274

[38] Huang Y. , Thörnqvist L., Ohlin M. Computational inference, validation, and analysis of 5’UTR-leader sequences of alleles of immunoglobulin heavy chain variable genes. Front. Immunol. 2021; 12:730105. 34671351 10.3389/fimmu.2021.730105 PMC8521166

[39] Lee J.H. , Toy L., Kos J.T., Safonova Y., Schief W.R., Havenar-Daughton C., Watson C.T., Crotty S. Vaccine genetics of IGHV1-2 VRC01-class broadly neutralizing antibody precursor naïve human B cells. NPJ Vaccines. 2021; 6:113. 34489473 10.1038/s41541-021-00376-7 PMC8421370

[40] Ralph D.K. , Matsen IV F.A. Consistency ofVDJ rearrangement and substitution parameters enables accurate B cell receptor sequence annotation. PLoS Comput. Biol. 2016; 12:e1004409. 26751373 10.1371/journal.pcbi.1004409 PMC4709141

[41] Musvosvi M. , Huang H., Wang C., Xia Q., Rozot V., Krishnan A., Acs P., Cheruku A., Obermoser G., Leslie A. et al. T cell receptor repertoires associated with control and disease progression following Mycobacterium tuberculosis infection. Nat. Med. 2023; 29:258–269. 36604540 10.1038/s41591-022-02110-9 PMC9873565

[42] Russell M.L. , Souquette A., Levine D.M., Schattgen S.A., Allen E.K., Kuan G., Simon N., Balmaseda A., Gordon A., Thomas P.G. et al. Combining genotypes and T cell receptor distributions to infer genetic loci determining V(D)J recombination probabilities. Elife. 2022; 11:e73475. 35315770 10.7554/eLife.73475 PMC8940181

[43] Pushparaj P. , Nicoletto A., Sheward D.J., Das H., Dopico X.C., Vidakovics L.P., Hanke L., Chernyshev M., Narang S., Kim S. et al. Immunoglobulin germline gene polymorphisms influence the function of SARS-CoV-2 neutralizing antibodies. Immunity. 2022; 56:193–206. 36574772 10.1016/j.immuni.2022.12.005 PMC9742198

[44] Boyd S.D. , Gaëta B.A., Jackson K.J., Fire A.Z., Marshall E.L., Merker J.D., Maniar J.M., Zhang L.N., Sahaf B., Jones C.D. et al. Individual variation in the germline Ig gene repertoire inferred from variable region gene rearrangements. J. Immunol. 2010; 184:6986–6992. 20495067 10.4049/jimmunol.1000445 PMC4281569

[45] Jackson K.J. , Kos J.T., Lees W., Gibson W.S., Smith M.L., Peres A., Yaari G., Corcoran M., Busse C.E., Ohlin M. et al. A BALB/c IGHV Reference Set, defined by haplotype analysis of long-read VDJ-C sequences from F1 (BALB/c/C57BL/6) mice. 2022; bioRxiv doi:01 March 2022, preprint: not peer reviewed 10.1101/2022.02.28.482396. PMC9205180 35720344

[46] Thörnqvist L. , Ohlin M. Critical steps for computational inference of the 3’-end of novel alleles of immunoglobulin heavy chain variable genes-illustrated by an allele of IGHV3-7. Mol. Immunol. 2018; 103:1–6. 30172112 10.1016/j.molimm.2018.08.018

[47] Watson C.T. , Kos J.T., Gibson W.S., Newman L., Deikus G., Busse C.E., Smith M.L., Jackson K.J., Collins A.M. A comparison of immunoglobulin IGHV, IGHD and IGHJ genes in wild-derived and classical inbred mouse strains. Immunol. Cell Biol. 2019; 97:888–901. 31441114 10.1111/imcb.12288

[48] Kaduk M. , Corcoran M., Karlsson Hedestam G.B. Addressing IGHV gene structural diversity enhances immunoglobulin repertoire analysis: lessons from rhesus macaque. Front. Immunol. 2022; 13:818440. 35419009 10.3389/fimmu.2022.818440 PMC8995469

[49] Bernat N.V. , Corcoran M., Nowak I., Kaduk M., Dopico X.C., Narang S., Maisonasse P., Dereuddre-Bosquet N., Murrell B., Hedestam G. B.K. Rhesus and cynomolgus macaque immunoglobulin heavy-chain genotyping yields comprehensive databases of germline VDJ alleles. Immunity. 2021; 54:355–366. 33484642 10.1016/j.immuni.2020.12.018

[50] Pennell M. , Rodriguez O.L., Watson C.T., Greiff V. The evolutionary and functional significance of germline immunoglobulin gene variation. Trends Immunol. 2022; 44:7–21. 36470826 10.1016/j.it.2022.11.001

[51] Avnir Y. , Tallarico A.S., Zhu Q., Bennett A.S., Connelly G., Sheehan J., Sui J., Fahmy A., Huang C.-Y., Cadwell G. et al. Molecular signatures of hemagglutinin stem-directed heterosubtypic human neutralizing antibodies against influenza A viruses. PLoS Pathog. 2014; 10:e1004103. 24788925 10.1371/journal.ppat.1004103 PMC4006906

[52] Avnir Y. , Watson C.T., Glanville J., Peterson E.C., Tallarico A.S., Bennett A.S., Qin K., Fu Y., Huang C.-Y., Beigel J.H. et al. IGHV1-69 polymorphism modulates anti-influenza antibody repertoires, correlates with IGHV utilization shifts and varies by ethnicity. Sci. Rep. 2016; 6:20842. 26880249 10.1038/srep20842 PMC4754645

[53] Chaisson M.J. , Sanders A.D., Zhao X., Malhotra A., Porubsky D., Rausch T., Gardner E.J., Rodriguez O.L., Guo L., Collins R.L. et al. Multi-platform discovery of haplotype-resolved structural variation in human genomes. Nat. Commun. 2019; 10:1784. 30992455 10.1038/s41467-018-08148-z PMC6467913

[54] Collins A.M. , Yaari G., Shepherd A.J., Lees W., Watson C.T. Germline immunoglobulin genes: disease susceptibility genes hidden in plain sight?. Curr. Opin. Syst. Biol. 2020; 24:100–108. 37008538 10.1016/j.coisb.2020.10.011 PMC10062056

