# Supplementary material for: IGHV allele similarity clustering improves genotype inference from adaptive immune receptor repertoire sequencing data
Source: Nucleic Acids Res. 2023 Aug 7;51(16):e86. doi: 10.1093/nar/gkad603 (PMC10484671; doi:10.1093/nar/gkad603)
Supplement: gkad603_Supplemental_File [file gkad603_supplemental_file.pdf]

IGHV allele similarity clustering improves genotype inference from adaptive immune receptor repertoire sequencing data

Ayelet Peres[1,2], William D. Lees[3], Oscar L. Rodriguez[4], Noah Y. Lee[5,6], Pazit Polak[1,2], Ronen Hope[1], Meirav Kedmi[6,7,8], Andrew M. Collins[9], Mats Ohlin[10], Steven H. Kleinstein[5,6], Corey T Watson[4] and Gur Yaari[1,2,†]

[1]Faculty of Engineering, Bar Ilan University, 5290002 Ramat Gan, Israel
[2]Bar Ilan Institute of Nanotechnology and Advanced Materials, Bar Ilan University, 5290002 Ramat Gan, Israel
[3]Institute of Structural and Molecular Biology, Birkbeck College, University of London, London, United Kingdom
[4]Department of Biochemistry and Molecular Genetics, University of Louisville School of Medicine, Louisville, KY, United States
[5]Program in Computational Biology & Bioinformatics, Yale University, New Haven, Connecticut, USA
[6]Department of Pathology, Yale School of Medicine, New Haven, Connecticut, USA
[7]Division of Hematology and Bone Marrow Transplantation, Chaim Sheba Medical Center, Tel-Hashomer, Israel
[8]Sackler School of Medicine, Tel-Aviv University, Tel-Aviv, Israel
[9]The Mina & Everard Goodman Faculty of Life Sciences, Bar-Ilan University, Israel
[10]School of Biotechnology and Biomedical Sciences, University of New South Wales, Sydney, Australia
[11]Department of Immunotechnology Lund University, Lund, Sweden
[†]To whom correspondence should be addressed. Tel: +972 3 7384625; Email: gur.yaari@biu.ac.il

June 26, 2023

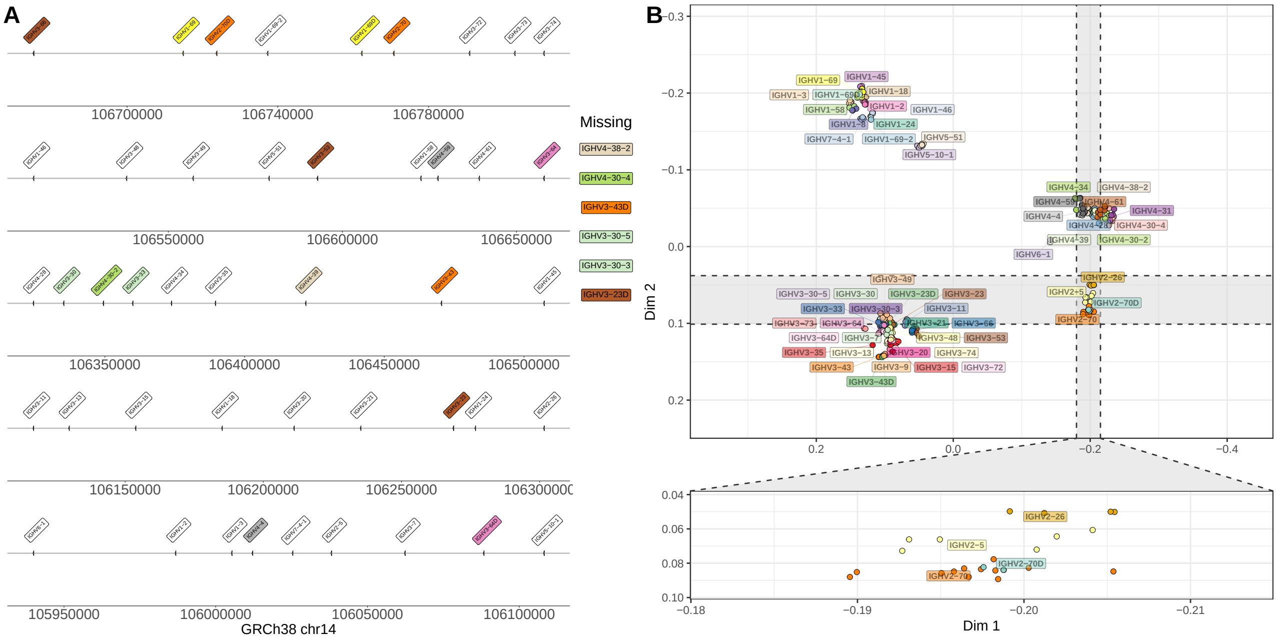

Figure S1: **Sequence similarity in the IGH locus.** (A) Illustration of the complex, repetitive nature of the IG heavy chain V (IGHV) locus on chromosome 1, based on the GRCh38 [2] assembly. The illustration shows that several DNA segments are duplicated, leading to the presence of identical allele sequences at different locations, for example IGHV2-70/IGHV2-70D, IGHV3-23/IGHV3-23D [3]. The x-axis is the IGHV locus on human chromosome 14, GRCh38 [2] coordinates. The colored genes (not dark gray) are those with 95%-100% germline sequence similarity, meaning these IUIS genes include alleles that appear in joint similarity clusters. Genes that share similarity but do not have a genomic location in the GRCh38 assembly, are shown to the right of the plot. Dark gray genes are stand-alone genes, and other colors indicate genes with similar alleles. (B) As previously shown for IGHV sequences [1], exploring the similarity between all full length functional alleles within a germline set shows that in some cases alleles from different genes are clustered together. Multidimensional scaling of the IGHV IMGT germline set pairs distance matrix; plot shows the first two dimensions. Each dot is a functional allele colored by gene. The bottom panel shows a zoom into the IGHV2 subgroup, demonstrating the proximity between alleles of the duplicated genes (IGHV2-70 and IGHV2-70D).

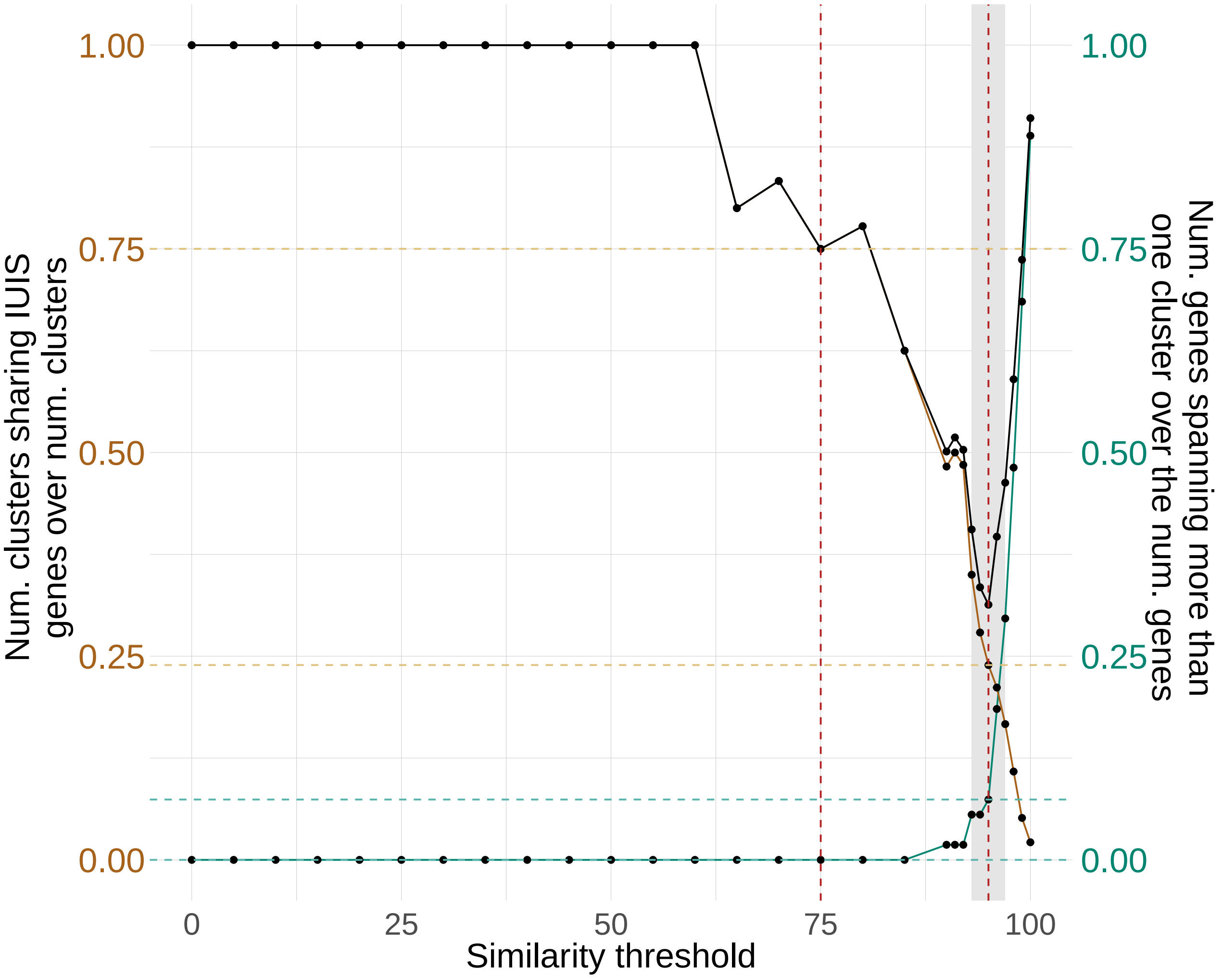

Figure S2: **ASCs similarity threshold** he axes show the number of genes spanning more than one cluster, normalized by the total number of genes, and the number of clusters that contain alleles from distinct genes, normalized by the total number of clusters, as a function of the similarity threshold. The x-axis is the similarity threshold. The left y-axis (yellow) is the number of clusters sharing a gene relative to the number of total clusters. The right y-axis (green) is the number of genes spanning more than one cluster, normalized by the total number of genes. The black line is the summation of the yellow and green line. The red dotted lines indicates the chosen similarity threshold, and the yellow and green dotted lines shows the respective y-axis values. The light gray strip indicates the thresholds bounds of 93% to 97%

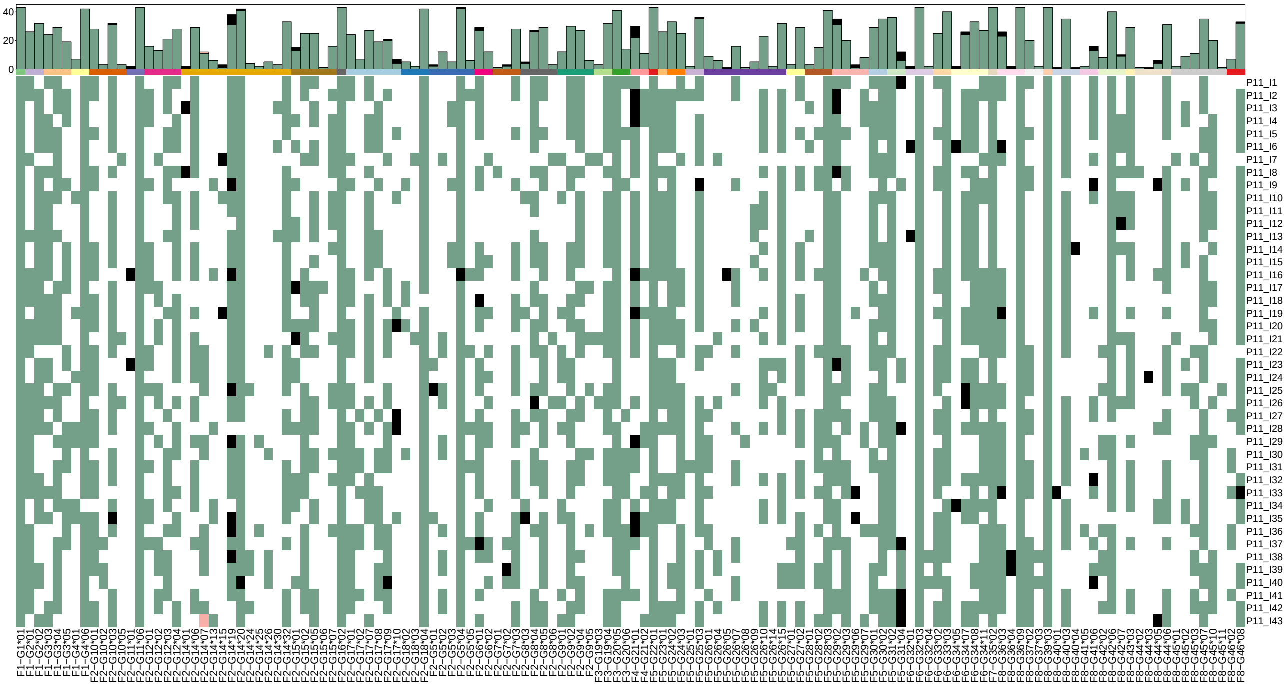

Figure S3: **P11 individuals genotype comparison.** A heatmap comparing the genotypes inferred from the gene-level and the allele-based method. The bottom panel is the heatmap comparison, where each row is a genotype inference of an individual from the P11 dataset and each column is a different allele. Black and Pink colors represent alleles that only entered the genotype either in the allele-based method or in gene-based method with a 12.5% threshold, respectively. Green represents alleles that entered the genotype in both methods, and white represents alleles that did not pass in both methods. The top panel is the summation of the heatmap events. The y-axis is the count of the individuals for which a given allele entered the genotype. The x-axis is the different alleles.

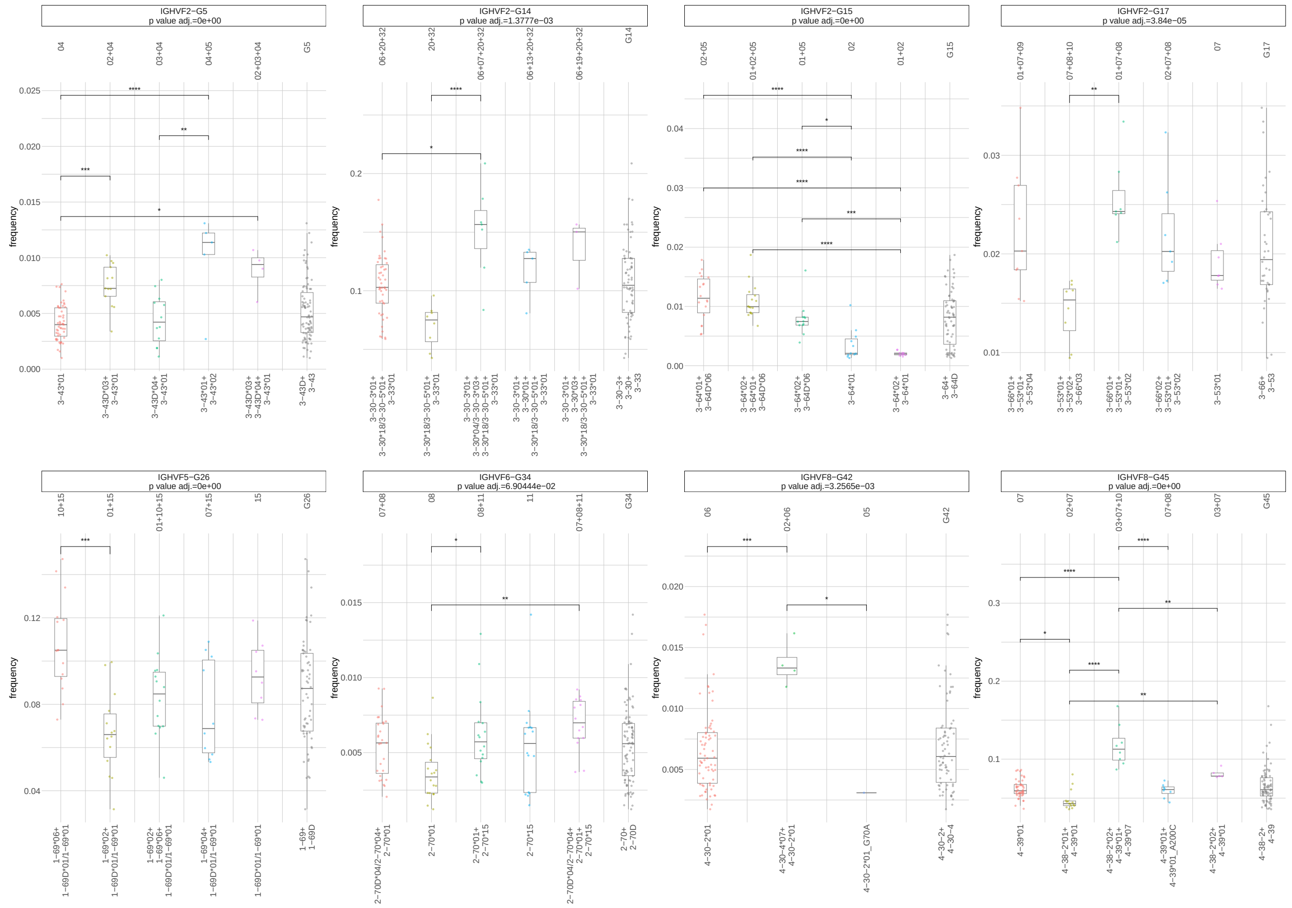

Figure S4: **Gene usage-based genotype.** The absolute usage frequency (calls out of the total repertoire size) of the top five allele combinations for the given clusters. The x-axis columns are the cluster's allele combination after genotype inference, ordered by the number of individuals carrying the combinations. The y-axis is the absolute frequency of the cluster within the total repertoire. Each point is an individual's absolute frequency. The colors represent the order of the combinations, where the combination which is present in most individuals is colored in red and so on. The gray color, last column in every x-axis facet, indicates the absolute frequency in terms of the whole cluster. For each cluster, an ANOVA test was calculated and the adjusted p value is presented in the cluster's plot title. A Tukey's HSD multiple comparison test was calculated with the adjusted p value, comparing between allele clusters indicated on the connecting line; only the statistically significant combination were drawn. ns: $p > 0.05$, *: $p <= 0.05$, **: $p <= 0.01$, ***: $p <= 0.001$, ****: $p <= 0.0001$

ASC Allele	IUIS Allele	Allele threshold
IGHVS1F1-G1*01	IGHV3-72*01	10^{-04}
IGHVS1F1-G2*01	IGHV3-73*01	10^{-04}
IGHVS1F1-G2*02	IGHV3-73*02	10^{-04}
IGHVS1F1-G3*01	IGHV3-49*02	10^{-04}
IGHVS1F1-G3*02	IGHV3-49*01	10^{-04}
IGHVS1F1-G3*03	IGHV3-49*05	10^{-04}
IGHVS1F1-G3*04	IGHV3-49*03	10^{-04}
IGHVS1F1-G3*05	IGHV3-49*04	10^{-04}
IGHVS1F1-G4*01	IGHV3-15*07	0.001
IGHVS1F1-G4*02	IGHV3-15*06	10^{-04}
IGHVS1F1-G4*03	IGHV3-15*05	0.001
IGHVS1F1-G4*04	IGHV3-15*04	0.001
IGHVS1F1-G4*05	IGHV3-15*02	10^{-04}
IGHVS1F1-G4*06	IGHV3-15*01	0.001
IGHVS1F1-G4*07	IGHV3-15*01_A313T	0.001
IGHVS1F1-G4*08	IGHV3-15*03	10^{-04}
IGHVS1F1-G4*09	IGHV3-15*08	10^{-04}
IGHVF2S1-G10*01	IGHV3-11*01	0.001
IGHVF2S1-G10*02	IGHV3-11*04	0.001
IGHVF2S1-G10*03	IGHV3-11*06	10^{-04}
IGHVF2S1-G10*04	IGHV3-11*03	10^{-04}
IGHVF2S1-G10*05	IGHV3-11*05	0.001
IGHVF2S1-G11*01	IGHV3-21*07	10^{-04}
IGHVF2S1-G11*02	IGHV3-21*05	0.001
IGHVF2S1-G11*03	IGHV3-21*06	0.001
IGHVF2S1-G11*04	IGHV3-21*04	0.001
IGHVF2S1-G11*05	IGHV3-21*03	0.001
IGHVF2S1-G11*06	IGHV3-21*01	0.001
IGHVF2S1-G11*07	IGHV3-21*02	0.001
IGHVF2S1-G12*01	IGHV3-48*03	0.001
IGHVF2S1-G12*02	IGHV3-48*04	0.001
IGHVF2S1-G12*03	IGHV3-48*01	0.001
IGHVF2S1-G12*04	IGHV3-48*02	0.001
IGHVF2S1-G13*01	IGHV3-35*02	10^{-04}
IGHVF2S1-G14*01	IGHV3-30-3*02	10^{-04}
IGHVF2S1-G14*02	IGHV3-30*04_C201T_G317A	0.001
IGHVF2S1-G14*03	IGHV3-30*08	10^{-04}
IGHVF2S1-G14*04	IGHV3-30*14	10^{-04}
IGHVF2S1-G14*05	IGHV3-30*09	10^{-04}
IGHVF2S1-G14*06	IGHV3-30-3*01	10^{-04}
IGHVF2S1-G14*07	IGHV3-30*04/IGHV3-30-3*03	0.001
IGHVF2S1-G14*08	IGHV3-30*17	10^{-04}
IGHVF2S1-G14*09	IGHV3-30*16	0.001
IGHVF2S1-G14*10	IGHV3-30*15	10^{-04}

IGHVF2S1-G14*11	IGHV3-30*11	10^{-}04
IGHVF2S1-G14*12	IGHV3-30*10	0.001
IGHVF2S1-G14*13	IGHV3-30*01	10^{-}04
IGHVF2S1-G14*14	IGHV3-30*07	0.001
IGHVF2S1-G14*15	IGHV3-30*20	10^{-}04
IGHVF2S1-G14*16	IGHV3-30*06	10^{-}04
IGHVF2S1-G14*17	IGHV3-30*19	10^{-}04
IGHVF2S1-G14*18	IGHV3-33*05	0.001
IGHVF2S1-G14*19	IGHV3-30*03	10^{-}04
IGHVF2S1-G14*20	IGHV3-30*18/IGHV3-30-5*01	10^{-}04
IGHVF2S1-G14*21	IGHV3-30*12	10^{-}04
IGHVF2S1-G14*22	IGHV3-30*05	10^{-}04
IGHVF2S1-G14*23	IGHV3-30*13	10^{-}04
IGHVF2S1-G14*24	IGHV3-30*02_G49A	0.001
IGHVF2S1-G14*25	IGHV3-30*02_A275G	0.001
IGHVF2S1-G14*26	IGHV3-30*02/IGHV3-30-5*02	0.001
IGHVF2S1-G14*27	IGHV3-33*02	10^{-}04
IGHVF2S1-G14*28	IGHV3-33*07	10^{-}04
IGHVF2S1-G14*29	IGHV3-33*04	10^{-}04
IGHVF2S1-G14*30	IGHV3-33*08	0.001
IGHVF2S1-G14*31	IGHV3-33*03	10^{-}04
IGHVF2S1-G14*32	IGHV3-33*01	10^{-}04
IGHVF2S1-G14*33	IGHV3-33*06	0.001
IGHVF2S1-G15*01	IGHV3-64*02	10^{-}04
IGHVF2S1-G15*02	IGHV3-64*01	10^{-}04
IGHVF2S1-G15*03	IGHV3-64*07	0.001
IGHVF2S1-G15*04	IGHV3-64*04	10^{-}04
IGHVF2S1-G15*05	IGHV3-64D*06	10^{-}04
IGHVF2S1-G15*06	IGHV3-64D*08	10^{-}04
IGHVF2S1-G15*07	IGHV3-64D*09	10^{-}04
IGHVF2S1-G15*08	IGHV3-64*03	10^{-}04
IGHVF2S1-G15*09	IGHV3-64*05	10^{-}04
IGHVF2S1-G16*01	IGHV3-74*03	0.001
IGHVF2S1-G16*02	IGHV3-74*01	10^{-}04
IGHVF2S1-G16*03	IGHV3-74*02	10^{-}04
IGHVF2S1-G17*01	IGHV3-66*01	0.001
IGHVF2S1-G17*01_G319C	IGHV3-66*04	0.001
IGHVF2S1-G17*02	IGHV3-66*02	10^{-}05
IGHVF2S1-G17*03	IGHV3-66*02_G303A	10^{-}04
IGHVF2S1-G17*04	IGHV3-53*03	10^{-}04
IGHVF2S1-G17*05	IGHV3-53*05	10^{-}04
IGHVF2S1-G17*06	IGHV3-53*02_C259T	0.001
IGHVF2S1-G17*07	IGHV3-53*01	0.001
IGHVF2S1-G17*08	IGHV3-53*02	0.001
IGHVF2S1-G17*09	IGHV3-53*04	10^{-}04

IGHVF2S1-G17*10	IGHV3-66*03	$10^{-}05$
IGHVF2S1-G18*01	IGHV3-23*02	$10^{-}04$
IGHVF2S1-G18*02	IGHV3-23*04	0.001
IGHVF2S1-G18*03	IGHV3-23*01_G239T	0.001
IGHVF2S1-G18*04	IGHV3-23D*01/IGHV3-23*01	0.001
IGHVF2S1-G18*05	IGHV3-23*03	$10^{-}04$
IGHVF2S1-G18*06	IGHV3-23*05	$10^{-}04$
IGHVF2S1-G5*01	IGHV3-43D*04_G4A	$10^{-}04$
IGHVF2S1-G5*02	IGHV3-43D*03	$10^{-}04$
IGHVF2S1-G5*03	IGHV3-43D*04	$10^{-}04$
IGHVF2S1-G5*04	IGHV3-43*01	$10^{-}04$
IGHVF2S1-G5*05	IGHV3-43*02	$10^{-}04$
IGHVF2S1-G6*01	IGHV3-20*01	$10^{-}04$
IGHVF2S1-G6*02	IGHV3-20*04	$10^{-}04$
IGHVF2S1-G7*01	IGHV3-9*04	$10^{-}04$
IGHVF2S1-G7*02	IGHV3-9*03	$10^{-}04$
IGHVF2S1-G7*03	IGHV3-9*01	0.001
IGHVF2S1-G7*04	IGHV3-9*02	0.001
IGHVF2S1-G8*01	IGHV3-13*02	$10^{-}04$
IGHVF2S1-G8*02	IGHV3-13*03	0.001
IGHVF2S1-G8*03	IGHV3-13*01_G290A_T300C	$10^{-}04$
IGHVF2S1-G8*04	IGHV3-13*05	$10^{-}04$
IGHVF2S1-G8*05	IGHV3-13*01	$10^{-}04$
IGHVF2S1-G8*06	IGHV3-13*04	$10^{-}04$
IGHVF2S1-G9*01	IGHV3-7*04	0.001
IGHVF2S1-G9*02	IGHV3-7*01	0.001
IGHVF2S1-G9*03	IGHV3-7*02	0.001
IGHVF2S1-G9*04	IGHV3-7*03	0.001
IGHVF2S1-G9*05	IGHV3-7*05	$10^{-}04$
IGHVF3S1-G19*01	IGHV5-10-1*02	$10^{-}04$
IGHVF3S1-G19*02	IGHV5-10-1*04	$10^{-}04$
IGHVF3S1-G19*03	IGHV5-10-1*01	0.001
IGHVF3S1-G19*04	IGHV5-10-1*03	0.001
IGHVF3S1-G20*01	IGHV5-51*02	$5x10^{-}04$
IGHVF3S1-G20*02	IGHV5-51*07	$5x10^{-}04$
IGHVF3S1-G20*03	IGHV5-51*06	$5x10^{-}04$
IGHVF3S1-G20*04	IGHV5-51*04	$5x10^{-}04$
IGHVF3S1-G20*05	IGHV5-51*01	$5x10^{-}04$
IGHVF3S1-G20*06	IGHV5-51*03	$5x10^{-}04$
IGHVF4S1-G21*01	IGHV7-4-1*01	$10^{-}05$
IGHVF4S1-G21*02	IGHV7-4-1*02	$10^{-}04$
IGHVF4S1-G21*03	IGHV7-4-1*04	$10^{-}04$
IGHVF4S1-G21*04	IGHV7-4-1*05	$10^{-}04$
IGHVF5S1-G22*01	IGHV1-24*01	$10^{-}04$
IGHVF5S1-G23*01	IGHV1-69-2*01	$10^{-}04$

IGHVF5S1-G24*01	IGHV1-58*03	0.001
IGHVF5S1-G24*02	IGHV1-58*01	10^{-04}
IGHVF5S1-G24*03	IGHV1-58*02	10^{-04}
IGHVF5S1-G25*01	IGHV1-45*03	10^{-05}
IGHVF5S1-G25*02	IGHV1-45*01	10^{-05}
IGHVF5S1-G25*03	IGHV1-45*02	10^{-05}
IGHVF5S1-G26*01	IGHV1-69*02	0.001
IGHVF5S1-G26*02	IGHV1-69*08	0.001
IGHVF5S1-G26*03	IGHV1-69*10	0.001
IGHVF5S1-G26*04	IGHV1-69*10_A54G	0.001
IGHVF5S1-G26*05	IGHV1-69*20	10^{-04}
IGHVF5S1-G26*06	IGHV1-69*09	0.001
IGHVF5S1-G26*07	IGHV1-69*04	0.001
IGHVF5S1-G26*08	IGHV1-69*04_T191C	0.001
IGHVF5S1-G26*09	IGHV1-69*17	0.001
IGHVF5S1-G26*10	IGHV1-69*06	0.001
IGHVF5S1-G26*11	IGHV1-69*06_G240A	0.001
IGHVF5S1-G26*12	IGHV1-69*19	10^{-04}
IGHVF5S1-G26*13	IGHV1-69*18	0.001
IGHVF5S1-G26*14	IGHV1-69*01_C26T	0.001
IGHVF5S1-G26*15	IGHV1-69D*01/IGHV1-69*01	0.001
IGHVF5S1-G26*16	IGHV1-69*14	0.001
IGHVF5S1-G26*17	IGHV1-69*13	0.001
IGHVF5S1-G26*18	IGHV1-69*05	0.001
IGHVF5S1-G26*19	IGHV1-69*12	0.001
IGHVF5S1-G26*20	IGHV1-69*16	10^{-04}
IGHVF5S1-G26*21	IGHV1-69*11	10^{-04}
IGHVF5S1-G26*22	IGHV1-69*15	0.001
IGHVF5S1-G27*01	IGHV1-8*03	0.001
IGHVF5S1-G27*02	IGHV1-8*01	10^{-04}
IGHVF5S1-G27*03	IGHV1-8*02	0.001
IGHVF5S1-G28*01	IGHV1-46*04	0.001
IGHVF5S1-G28*02	IGHV1-46*03	0.001
IGHVF5S1-G28*03	IGHV1-46*01	0.001
IGHVF5S1-G28*04	IGHV1-46*02	10^{-04}
IGHVF5S1-G29*01	IGHV1-2*07	10^{-04}
IGHVF5S1-G29*02	IGHV1-2*04	10^{-04}
IGHVF5S1-G29*03	IGHV1-2*02	0.001
IGHVF5S1-G29*04	IGHV1-2*03	10^{-04}
IGHVF5S1-G29*05	IGHV1-2*01	10^{-04}
IGHVF5S1-G29*06	IGHV1-2*05	10^{-04}
IGHVF5S1-G29*07	IGHV1-2*06	0.001
IGHVF5S1-G30*01	IGHV1-18*04	0.001
IGHVF5S1-G30*02	IGHV1-18*01	0.001
IGHVF5S1-G30*03	IGHV1-18*03	10^{-04}

IGHVF5S1-G31*01	IGHV1-3*05	0.001
IGHVF5S1-G31*02	IGHV1-3*01	$10^{-}05$
IGHVF5S1-G31*03	IGHV1-3*04	0.001
IGHVF5S1-G31*04	IGHV1-3*02	$10^{-}05$
IGHVF5S1-G31*05	IGHV1-3*03	$10^{-}04$
IGHVF6S1-G32*01	IGHV2-26*04	$10^{-}04$
IGHVF6S1-G32*02	IGHV2-26*03	$10^{-}04$
IGHVF6S1-G32*03	IGHV2-26*01	$10^{-}04$
IGHVF6S1-G32*04	IGHV2-26*02	$10^{-}04$
IGHVF6S1-G33*01	IGHV2-5*08	0.001
IGHVF6S1-G33*02	IGHV2-5*01	0.001
IGHVF6S1-G33*03	IGHV2-5*02	$5x10^{-}04$
IGHVF6S1-G33*04	IGHV2-5*09	$10^{-}04$
IGHVF6S1-G33*05	IGHV2-5*05	0.001
IGHVF6S1-G33*06	IGHV2-5*06	$10^{-}04$
IGHVF6S1-G34*01	IGHV2-70*12	$10^{-}04$
IGHVF6S1-G34*02	IGHV2-70*10	$10^{-}04$
IGHVF6S1-G34*03	IGHV2-70D*14	$10^{-}04$
IGHVF6S1-G34*04	IGHV2-70*16	$10^{-}04$
IGHVF6S1-G34*05	IGHV2-70*17	$10^{-}04$
IGHVF6S1-G34*06	IGHV2-70*04_A14G	$10^{-}04$
IGHVF6S1-G34*07	IGHV2-70D*04/IGHV2-70*04	$10^{-}04$
IGHVF6S1-G34*08	IGHV2-70*01	$10^{-}04$
IGHVF6S1-G34*09	IGHV2-70*13	0.001
IGHVF6S1-G34*10	IGHV2-70*11	$10^{-}04$
IGHVF6S1-G34*11	IGHV2-70*15	$10^{-}04$
IGHVF6S1-G34*12	IGHV2-70*18	$10^{-}04$
IGHVF6S1-G34*13	IGHV2-70*19	$10^{-}04$
IGHVF6S1-G34*14	IGHV2-70*20	$10^{-}04$
IGHVF7S1-G35*01	IGHV6-1*02	$5x10^{-}04$
IGHVF7S1-G35*02	IGHV6-1*01	$5x10^{-}04$
IGHVF7S1-G35*03	IGHV6-1*01_T91C	$5x10^{-}04$
IGHVF8S1-G36*01	IGHV4-4*08	$10^{-}04$
IGHVF8S1-G36*02	IGHV4-4*09	$10^{-}04$
IGHVF8S1-G36*03	IGHV4-59*08	$10^{-}04$
IGHVF8S1-G36*04	IGHV4-59*12	0.001
IGHVF8S1-G36*05	IGHV4-59*13	$10^{-}04$
IGHVF8S1-G36*06	IGHV4-59*11	0.001
IGHVF8S1-G36*07	IGHV4-59*07	$10^{-}04$
IGHVF8S1-G36*08	IGHV4-59*02	0.005
IGHVF8S1-G36*09	IGHV4-59*01	$10^{-}04$
IGHVF8S1-G36*10	IGHV4-59*01_G267A	$10^{-}04$
IGHVF8S1-G37*01	IGHV4-59*10	$10^{-}04$
IGHVF8S1-G37*02	IGHV4-4*07	$10^{-}04$
IGHVF8S1-G37*03	IGHV4-4*07_A70G	$10^{-}04$

IGHVF8S1-G38*01	IGHV4-34*09	$10^{-}04$
IGHVF8S1-G38*02	IGHV4-34*10	$10^{-}04$
IGHVF8S1-G39*01	IGHV4-34*11	$10^{-}04$
IGHVF8S1-G39*02	IGHV4-34*12	0.001
IGHVF8S1-G39*03	IGHV4-34*01	$10^{-}04$
IGHVF8S1-G39*04	IGHV4-34*02	0.002
IGHVF8S1-G39*05	IGHV4-34*04	$10^{-}04$
IGHVF8S1-G39*06	IGHV4-34*05	$10^{-}04$
IGHVF8S1-G40*01	IGHV4-4*01	$10^{-}04$
IGHVF8S1-G40*02	IGHV4-4*03	0.001
IGHVF8S1-G40*03	IGHV4-4*02	$10^{-}04$
IGHVF8S1-G40*04	IGHV4-4*10	$10^{-}04$
IGHVF8S1-G41*01	IGHV4-28*02	$10^{-}05$
IGHVF8S1-G41*02	IGHV4-28*06	$10^{-}05$
IGHVF8S1-G41*03	IGHV4-28*04	$10^{-}05$
IGHVF8S1-G41*04	IGHV4-28*07	$10^{-}05$
IGHVF8S1-G41*05	IGHV4-28*05	$10^{-}05$
IGHVF8S1-G41*06	IGHV4-28*01	$10^{-}05$
IGHVF8S1-G41*07	IGHV4-28*03	$10^{-}05$
IGHVF8S1-G42*01	IGHV4-30-2*03	$10^{-}04$
IGHVF8S1-G42*02	IGHV4-30-4*07	$10^{-}04$
IGHVF8S1-G42*03	IGHV4-30-2*05	$10^{-}04$
IGHVF8S1-G42*04	IGHV4-30-2*06	$5x10^{-}04$
IGHVF8S1-G42*05	IGHV4-30-2*01_G70A	$10^{-}04$
IGHVF8S1-G42*06	IGHV4-30-2*01	$10^{-}04$
IGHVF8S1-G42*07	IGHV4-30-2*01_C285T	$10^{-}04$
IGHVF8S1-G43*01	IGHV4-30-4*02	$10^{-}04$
IGHVF8S1-G43*02	IGHV4-30-4*01_A70G_A107G	$10^{-}04$
IGHVF8S1-G43*03	IGHV4-30-4*01	0.001
IGHVF8S1-G43*04	IGHV4-30-4*08	0.001
IGHVF8S1-G44*01	IGHV4-31*10	$10^{-}04$
IGHVF8S1-G44*02	IGHV4-31*11_G4C_G21C_C25T_A113C	$10^{-}04$
IGHVF8S1-G44*03	IGHV4-31*11	$10^{-}04$
IGHVF8S1-G44*04	IGHV4-31*02	0.001
IGHVF8S1-G44*05	IGHV4-31*01	$10^{-}04$
IGHVF8S1-G44*06	IGHV4-31*03	$10^{-}05$
IGHVF8S1-G45*01	IGHV4-38-2*02_G246A	0.001
IGHVF8S1-G45*02	IGHV4-38-2*01	$10^{-}04$
IGHVF8S1-G45*03	IGHV4-38-2*02	0.001
IGHVF8S1-G45*04	IGHV4-39*08	$10^{-}04$
IGHVF8S1-G45*05	IGHV4-39*02	$10^{-}04$
IGHVF8S1-G45*06	IGHV4-39*01_G315A	0.005
IGHVF8S1-G45*07	IGHV4-39*01	0.001
IGHVF8S1-G45*08	IGHV4-39*01_A200C	0.001

IGHVF8S1-G45*09	IGHV4-39*06	$10^{-}04$
IGHVF8S1-G45*10	IGHV4-39*07	0.001
IGHVF8S1-G45*11	IGHV4-39*09	$10^{-}04$
IGHVF8S1-G46*01	IGHV4-61*09	$5x10^{-}04$
IGHVF8S1-G46*02	IGHV4-61*02	0.001
IGHVF8S1-G46*03	IGHV4-61*11	$5x10^{-}04$
IGHVF8S1-G46*04	IGHV4-61*05	$10^{-}04$
IGHVF8S1-G46*05	IGHV4-61*10	$10^{-}04$
IGHVF8S1-G46*06	IGHV4-61*08	0.001
IGHVF8S1-G46*07	IGHV4-61*03	$10^{-}04$
IGHVF8S1-G46*08	IGHV4-61*01	$10^{-}04$
IGHVF8S1-G46*09	IGHV4-61*01_A41G	$10^{-}04$

Table S1: **Allele cluster reference table.** The table contains the allele clusters for the full-length sequence germline set (S1) shown in Figure 2. The columns describe the alleles names according to the allele clusters naming scheme and the matching IUIS allele. The first column is the family, the second column is the allele cluster, the third column is the allele name, and the fourth column is the IUIS matching allele.

References

[1] Shishi Luo, Jane A Yu, and Yun S Song. Estimating copy number and allelic variation at the immunoglobulin heavy chain locus using short reads. *PLoS computational biology*, 12(9):e1005117, 2016.

[2] Valerie A. Schneider, Tina Graves-Lindsay, Kerstin Howe, Nathan Bouk, Hsiu-Chuan Chen, Paul A. Kitts, Terence D. Murphy, Kim D. Pruitt, Françoise Thibaud-Nissen, Derek Albracht, Robert S. Fulton, Milinn Kremitzki, Vince Magrini, Chris Markovic, Sean McGrath, Karyn Meltz Steinberg, Kate Auger, Will Chow, Joanna Collins, Glenn Harden, Tim Hubbard, Sarah Pelan, Jared T. Simpson, Glen Threadgold, James Torrance, Jonathan Wood, Laura Clarke, Sergey Koren, Matthew Boitano, Heng Li, Chen-Shan Chin, Adam M. Phillippy, Richard Durbin, Richard K. Wilson, Paul Flicek, and Deanna M. Church. Evaluation of GRCh38 and de novo haploid genome assemblies demonstrates the enduring quality of the reference assembly. *bioRxiv*, 2016.

[3] Corey T Watson, Karyn M Steinberg, John Huddleston, Rene L Warren, Maika Malig, Jacqueline Schein, A Jeremy Willsey, Jeffrey B Joy, Jamie K Scott, Tina A Graves, Richard K Wilson, Robert A Holt, Evan E Eichler, and Felix Breden. Complete haplotype sequence of the human immunoglobulin heavy-chain variable, diversity, and joining genes and characterization of allelic and copy-number variation. *The American Journal of Human Genetics*, 92(4):530–546, 2013.
